# Effect of arsenate substitution on phosphate repository of cell: a computational study

**DOI:** 10.1098/rsos.181565

**Published:** 2018-11-21

**Authors:** Amit Singh, Kousik Giri

**Affiliations:** Department of Computational Sciences, Central University of Punjab, Bathinda-151001, India

**Keywords:** quantum mechanics/molecular mechanics, DNA modelling, arsenate hydrolysis, phosphate hydrolysis, quantum calculations

## Abstract

The structural analogy with phosphate derives arsenate into various metabolic processes associated with phosphate inside the organisms. But it is difficult to evaluate the effect of arsenate substitution on the stability of individual biological phosphate species, which span from a simpler monoester form like pyrophosphate to a more complex phosphodiester variant like DNA. In this study, we have classified the physiological phosphate esters into three different classes on the basis of their structural differences. This classification has helped us to present a concise theoretical study on the kinetic stability of phosphate analogue species of arsenate against hydrolysis. All the calculations have been carried out using QM/MM methods of our Own N-layer Integrated molecular Orbital molecular Mechanics (ONIOM). For quantum mechanical region, we have used M06-2X density functional with 6-31+G(2d,2p) basis set and for molecular mechanics we have used the AMBER force field. The calculated rate constants for hydrolysis show that none of the phosphate analogue species of arsenate has a reasonable stability against hydrolysis.

## 1. Introduction

Maintaining negative charge at physiological pH leads phosphate (PO${\;}_{4}^{2-}$ or P_*i*_) ester to dominate the living world as it prevents chemically stored energy and genetic material from escaping the cell [1]. (V) is also capable of retaining negative charge in its phosphate analogue arsenate (AsO${\;}_{4}^{2-}$ or As_*i*_) over a range of physiological pH conditions [2] and it is already demonstrated in bacteria that at a high concentration ratio of As_*i*_/P_*i*_ the phosphate transporters allow the transport of As_*i*_ on the basis of structural similarity [3]. This structural analogy further leads to the integration of As_*i*_ into various metabolic processes associated with P_*i*_ inside the organisms [2], whereas the primary concern related to the P_*i*_ analogue species of As_*i*_ (PASA) is their rapid rate of hydrolysis [1]. However, the scientific world is really surprised by the discovery of arsenate substituted DNA backbone in place of phosphate (As_*i*_-DNA) in a bacterium strain GFAJ-1 of the Halomonadaceae family, which was isolated from Mono Lake, California with a very high As_*i*_/P_*i*_ ratio (greater than 103) [4]. The theoretical investigations on the possibility of As_*i*_-DNA also revealed that both arsenodiester and phosphodiester linkages have similar geometric and conformational properties [5,6]. Although these theoretical studies support geometrical similarities between As_*i*_-DNA and P_*i*_-DNA, neither of them claims the stability of As_*i*_-DNA against hydrolysis. Whereas the models described by Fekry *et al.* and Mládek *et al.* to estimate the kinetic stability of phosphodiester and arsenodiester linkages in DNA lack the fundamental base stacking interactions, also called *π* − *π* stacking [6,7]. In order to evaluate the stability of a molecule like DNA, a more realistic model is required because the work of Wang *et al*. reveals that the stacking between neighbouring bases in DNA single strands raises the activation energy barrier for hydrolysis of DNA [8]. Along with this, it is found that the discovery of As_*i*_-DNA in GFAJ-1 captured the attention of the scientific community from other PASA. However, it is not clear how As_*i*_ could be directly incorporated into P_*i*_-DNA because neither As_*i*_ nor P_*i*_ are the substrates for the DNA synthesizing enzymes and neither of these are directly incorporated into the diester backbone of the DNA. There are various metabolic steps through which monosaccharides are processed using P_*i*_ biomolecules and metabolized into nucleoside triphosphate (NTP’s). NTP’s are the monomeric units of nucleic acid polymers (DNA and RNA), and P_*i*_ is able to form the phosphodiester backbone of the DNA through the nucleophilic substitution reaction between deoxy-NTP molecules. Similarly, before reaching DNA, As_*i*_ must have a reasonable stability in its dNTP analogues, whereas biosynthesis of As_*i*_ analogue of dNTP again depends upon the availability of some other PASA like Ribose-5-As_*i*_, Glucose-1-As_*i*_, Pyro-As_*i*_, etc. [9]. Physiologically, these are dianionic monoesters of P_*i*_ and highly sensitive to As_*i*_ substitution [10]. Hence, to investigate the effect of As_*i*_ substitution on the P_*i*_ repository of the cell a comparative study at different biomolecular level is needed, that is not merely confined to analysing the impact of As_*i*_ substitution on the stability of a particular P_*i*_ biomolecule. However, due to a vast number of P_*i*_ species inside organisms, it is difficult to compare the rate of hydrolysis for each and every PASA against their respective P_*i*_ analogue. In this study, through a unique classification of P_*i*_ biomolecules, we examined the consequence of As_*i*_ substitution on the kinetic stability of P_*i*_ species spanning from a simpler monoester form like pyrophosphate to a more complex phosphodiester variant like DNA.

### 1.1. Hydrolysis pathways

**Figure 1.**
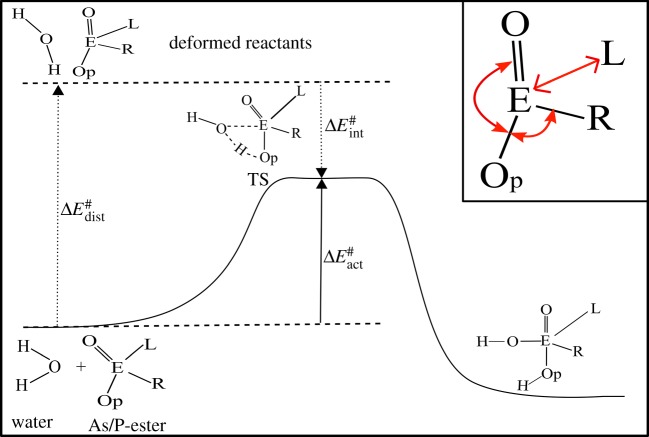
The initial step of associative pathway for the hydrolysis of (As/P) esters. Inset showing major geometrical distortion (in red) observed in ester geometry. (*E* = As/P, *L* = leaving group, O_*p*_ = oxygen atom of ester capturing proton from water, $\Delta E_{\mathrm{dist}}^{\#}=\text{distortion}$ energy, $\Delta E_{\mathrm{int}}^{\#}=\text{interaction}$ energy, $\Delta E_{\mathrm{act}}^{\#}=\text{activation}$ energy).

Pathways of the reaction for the hydrolysis of As_*i*_ and P_*i*_ are well documented in the literature [11–13]. The associative pathway is suggested to be dominant for both the esters [11] and follows the S_*N*_2 mechanism with first-order kinetics. The initial step of the pathway as shown in figure 1 involves the attack of water as a nucleophile on the central atom (As/P) of the ester. It is found that the attack of water has the highest barrier in terms of activation energy and hence determines the rate of hydrolysis of the ester [6,11,12]. In addition to the nature of reactant and attacking nucleophile, the chemistry of the leaving group is also the key factor for the kinetics of nucleophilic substitution reactions. However, the complete mechanism of the reaction for the nucleophilic attack on different types of phosphate ester was investigated in detail by Lopez *et al.* who concluded that the rate determining step is always that for the attack of nucleophile rather than the departure of the leaving group [12]. In other words, the activation energy barrier for nucleophilic substitution is mainly influenced by attacking nucleophile and steric congestion around a central atom [14,15]. We employed the activation strain model [14] to relate the height of the activation energy barrier with the geometrical deformation and rigidity of reactants (figure 1). Our study aims at comparing the kinetic stability of As and P esters against water, hence we only modelled the reactants and transition state (TS) structures which belong to the rate determining step. In an attempt to understand the TS, it was found that after reaching the TS for monoanionic monoesters and diesters of P form a reaction intermediate, having a pentacoordinated centre with trigonal bipyramidal geometry [12]. However, direct product is observed in the case of dianionic monoester of P [12]. We also observed that the TS is followed by a pentacovalent intermediate, during the hydrolysis of all classes of PASA, irrespective of the type of ester and anionic form. The reaction via pentacovalent intermediate is further proceeded by internal proton transfer that leads to the breaking of the As/P-O bond [6,11,12]. The structures of reactant and TS as shown in figure 1 for all three classes were modelled (available with the electronic supplementary material). We carried out intrinsic reaction coordinate calculations (movie files available with the electronic supplementary material) to confirm coordinates of the reactant and intermediate for the initial step as shown in figure 1.

### 1.2. Classification

In order to determine the effect of As_*i*_ substitution on the P_*i*_ repository of the cell, we first need to understand the physiological functions of P_*i*_ species. P_*i*_ has a variety of roles inside a cell, and is incorporated into biomolecules mainly through kinases. This process is also known as phosphorylation and the reverse of this is de-phosphorylation, removal of P_*i*_ by hydrolases. This cycle of addition and removal of P_*i*_ governs biological phenomena such as activation or deactivation of proteins, synthesis of DNA and removing falsely paired nucleotide bases from it, breaking down hexoses into trioses, synthesis and utilization of ATP, etc. However, all of these happen at specific sites in a well-controlled way. Phosphorylation/de-phosphorylation on proteins usually takes place on the amino acids that have a hydroxyl group on their side chains, e.g. serine, threonine and tyrosine. Similarly, on hexoses the hydroxyl group at terminal carbon atoms is the site of phosphorylation/de-phosphorylation. Whereas in ADP, the hydroxyl group attached to the terminal beta phosphorus (P*β*) atom is used to synthesise ATP [9]. These specific sites are conserved throughout the organisms, and the rate of phosphorylation/de-phosphorylation at these sites is well synchronized with each biological process. We classified physiological P_*i*_ species based on the type of ester and conserved sites through which P_*i*_ anchors with biomolecules (table 1). The same sites also behaved as leaving groups during dephosphorylation or hydrolysis of P_*i*_ ester.

**Table 1.** Different classes of PASA.

class	ester type	leaving group	example of PASA	P_*i*_–analogue
1A	monoester	-O-P-R_1_	Pyro-As_*i*_	Pyro-P_*i*_
1B	monoester	-O-C-R_2_	Ribose-1-As_*i*_	Ribose-1-P_*i*_
2	diester	-O-C-R_3_	As_*i*_-DNA	P_*i*_-DNA

**Figure 2.**
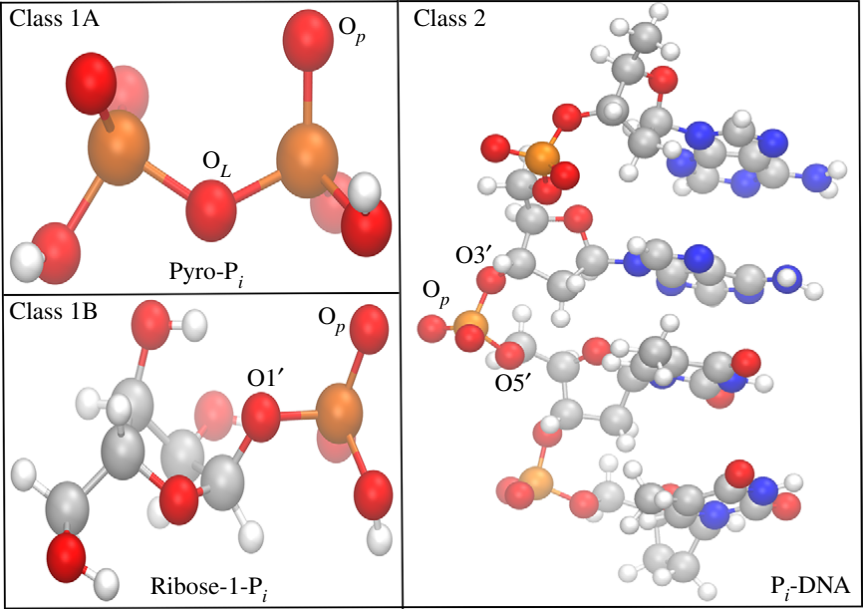
P_*i*_ biomolecules modelled for each class. Key atoms are labelled to compare the geometry of reactant with TS structure. Atoms of P, O, C, N and H coloured in orange, red, grey, blue and white, respectively. Where O_*L*_ = oxygen atom leaving during hydrolysis, O_*p*_ = oxygen atom capturing proton from water molecule, Oi′ = oxygen atom attached to Ci′ ring atom of ribose.

The modelled compound from each class is shown in figure 2. Our classification broadly covers most of the P_*i*_ biomolecules, Class 1A representing the energy currency of the cell, like ATP and ADP, while examples of Class 1B members are sugar or glycerol moiety containing biomolecules like Glucose-1-P_*i*_, Fructose-1,6-Bi-P_*i*_, Phosphoglycerate, etc. Whereas the molecules capable of storing genetic information belong to Class 2, like DNA and RNA. This classification not only enabled us to determine the relative stability of PASAs against hydrolysis with their respective P_*i*_ analogue, it also helped us to understand the consequence of As_*i*_ substitution at two different levels of biological esters (monoester and diester) and the role of conserved sites for phosphorylation/de-phosphorylation in the kinetics of hydrolysis.

## 2. Methods

For quantum chemical calculations, we need a suitable method to describe the thermochemical kinetics accurately for hydrolysis of inorganic systems like pyro-P_*i*_/As_*i*_ and for bigger biomolecular systems like DNA that have non-covalent interactions. Zhao and Truhlars’ work recommended Minnesota density functionals for thermochemistry, thermochemical kinetics and non-covalent interactions [16]. The presence of a polar solvent (*ɛ* = 78.4, water) was mimicked with the polarizable continuum model using the integral equation formalism variant [17]. We carried out computations using M06-2X Minnesota density functional with a 6-31+G(2d,2p) basis set for the hydrolysis of pyroarsenate, and calculated the rate of the hydrolysis at 298 K using equation (2.1)

2.1 $$k(T)=\frac{k_{B}T}{hc^{\circ }}\;{\text{e}}^{-\Delta G^{†\circ }/RT},$$

where *c*° = 1 and $\Delta G^{†\circ }$ represents the free energy of activation. The calculated rate constant value of 0.08 s^−1^ is in good agreement with the available experimental value of 0.05 s^−1^ for the non-enzymatic hydrolysis of pyroarsenate. [18]

### 2.1. Modelling of P_*i*_-DNA and As_*i*_-DNA

**Figure 3.**
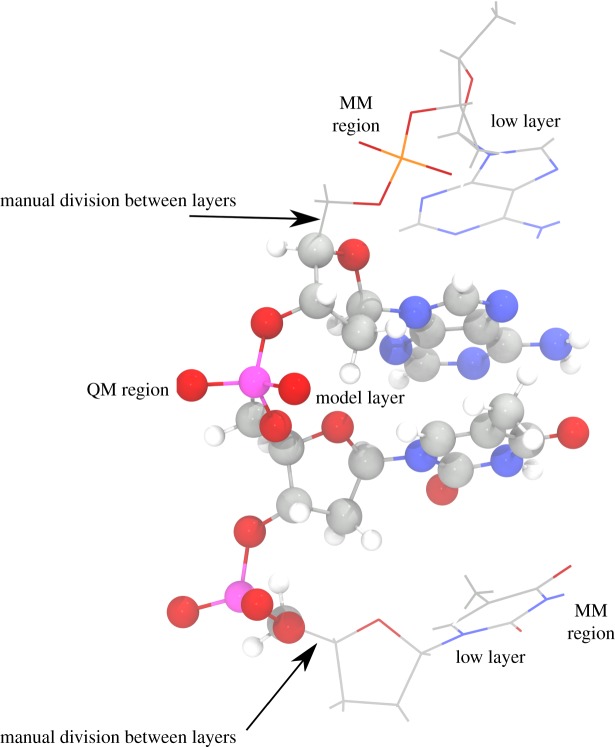
Scheme of dividing As_*i*_-DNA for two-layer ONIOM. Atoms in QM region are shown by ball and stick representation whereas atoms in Low MM region are shown by wireframe. Atoms of As, O, C, N, P and H coloured in purple, red, grey, blue, orange and white, respectively.

The crystal structure of B-DNA dodecamer on PDB (id: 1bna.pdb) was used to construct a single stranded P_*i*_-DNA model system consisting of d(TpTpApA) nucleotide bases. For modelling the As_*i*_-DNA d(TAsTAsApA) structure, we replaced the respective two phosphorus atoms of P_*i*_-DNA d(TpTpApA) with arsenic. It is difficult to deal with the whole system quantum mechanically (QM) due to the size complexity of P_*i*_-DNA and As_*i*_-DNA. Hence, QM was only applied on the portion which is involved in the reaction and the remaining part of the structure was dealt with using molecular mechanics (MM). We used two layers (our Own N-layer Integrated molecular Orbital molecular Mechanics) ONIOM [19], integrated into a Gaussian 09 [20] package, for QM/MM calculations. To avoid the problem of cancellation in ONIOM, a non-polar bond between two sp^3^ hybridized C atoms, which is more than four bonded atoms away from the centre of the reaction, was selected to divide the structure into model layer (QM region) and low layer (MM region) as shown in figure 3.

**Figure 4.**
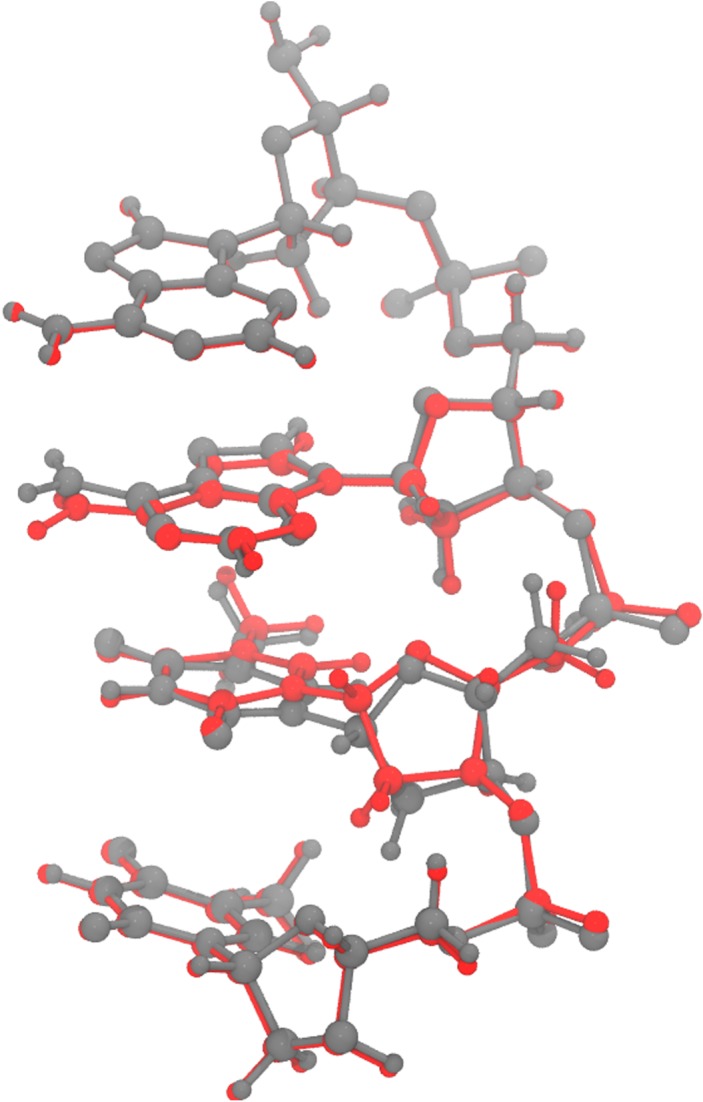
Superimposition of our modelled P_*i*_-DNA (in red) with Minnesota density functional M06-2X 6-31+G(2d,2p)/AMBER geometry optimization over the identical portion of B-DNA crystal structure (in grey) with a rmsd value of 1.54 ångstrom.

The MM region was treated with AMBER [21] and frozen to maintain the structural constraints for diester backbone. For the QM region we chose M06-2X with 6-31+G(2d,2p) basis set which provides a good description of base stacking interaction for single-stranded DNA [22]. Our modelled P_*i*_-DNA with M06-2X 6-31+G(2d,2p)/AMBER optimization of the geometry shows a reasonable geometrical similarity with the identical portion of B-DNA crystal structure with a RMSD value of 1.54 Å as shown in figure 4.

## 3. Results and discussion

**Figure 5.**
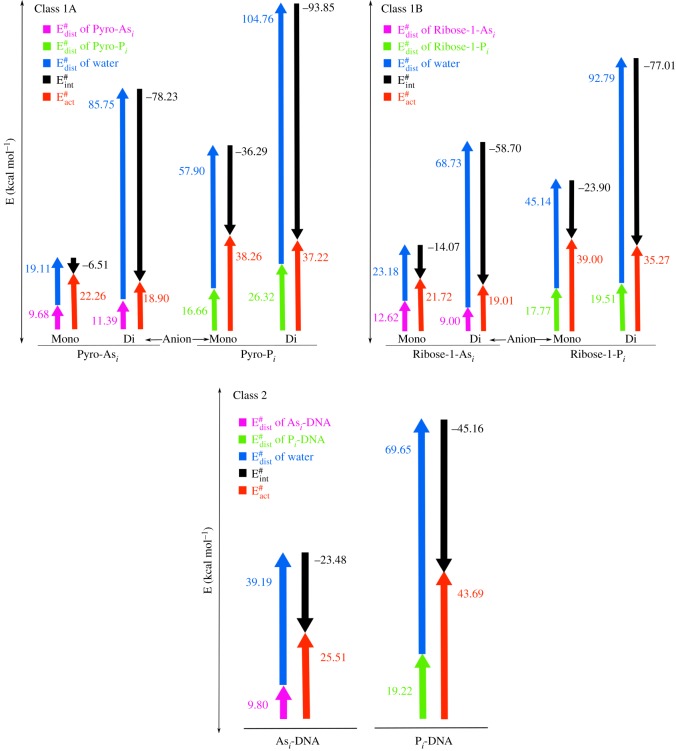
Graph of distortion, interaction and activation energies for the rate determining step of reaction between As_*i*_/P_*i*_ esters and water. (green: distortion energy, blue: distortion energy, red: activation energy, black: interaction energy, in kcal mol^−1^).

The oxygen atoms bonded to central atom (As/P) of the esters are labelled (figure 2) to discuss and compare the parameters of the geometry between modelled reactants and TS compounds. Comparison of the main geometrical data between computed arsenate and phosphate models belong to Class 1A, 1B and 2 is presented in tables 2–4, respectively. Although the tabular data shows differences among the selected bonded parameters of the reactant and TS structures, these parameters were rarely used to relate the two different ester species. However, using the activation strain model we represented these geometrical changes in terms of the following energy terms ($\Delta E_{\mathrm{dist}}^{\#}=\text{distortion}$ energy, $\Delta E_{\mathrm{int}}^{\#}=\text{interaction}$ energy, $\Delta E_{\mathrm{act}}^{\#}=\text{activation}$ energy), were basically derived from single point energy calculations [14]. The calculated values for distortion, interaction and activation energies for each species of three classes were shown in figure 5. Throughout the classification, irrespective of the anionic form, geometry of P_*i*_ esters were more deformed in TS compared to the As_*i*_ esters, which is reflected in the values of distortion energy (green bars) in figure 5. The higher deformity in TS for P_*i*_ esters might be due to the smaller size of the central P atom, compared to the bigger size of the As atom which allows more space for the incoming water nucleophile. Similarly, we also found that the geometry of dianionic P_*i*_ esters was more deformed than its monoionic form because two negatively charged oxygen atoms on the smaller central P atom possess a higher steric barrier for incoming nucleophile. However, the effect of higher deformity in the geometry of dianionic P_*i*_ monoester was not reflected in terms of the activation energy barrier due to the higher contribution from the interaction energy. In fact, it is the interaction energy which lowers the activation energy barrier for the hydrolysis of all the dianionic As_*i*_ as well as P_*i*_ monoesters. Interestingly, in the case of diester of class 2, the activation energy barrier achieved a comparable height equal to the interaction energy and this signifies the importance of diester linkages inside a living system. Compared to As_*i*_-DNA a lot of energy is required to deform the structure of P_*i*_-DNA during hydrolysis and this causes the biology to rely upon the smaller P atom for more rigid and stable geometry compared to the larger As atom. Apart from the comparison of geometrical deformity, the kinetics of hydrolysis for the esters is also discussed further in detail.

**Table 2.** Geometrical values of the Class 1A modelled compounds obtained from M06-2X 6-31+G(2d,2p) computation (R, reactant; TS, transition state).

	monoester				diester			
geometric	Pyro-P_*i*_		Pyro-As_*i*_		Pyro-P_*i*_		Pyro-As_*i*_	
parameters	R	TS	R	TS	R	TS	R	TS
d(E-O_*L*_) Å	1.64	1.68	1.76	1.77	1.70	1.79	1.81	1.82
d(E-O_*p*_) Å	1.48	1.58	1.63	1.70	1.51	1.65	1.66	1.75
$∠({\text{O}}_{L}$-E-O_*p*_)°	108.27	93.39	109.65	99.13	105.41	87.03	102	91.63

**Table 3.** Geometrical values of the Class 1B modelled compounds obtained from M06-2X 6-31+G(2d,2p) computation.

	monoester				diester			
geometric	Ribose -1-P_*i*_		Ribose-1-As _*i*_		Ribose-1-P_*i*_		Ribose-1-As_*i*_	
parameters	R	TS	R	TS	R	TS	R	TS
d(E-O1′) Å	1.63	1.67	1.76	1.79	1.70	1.74	1.80	1.81
d(E-O_*p*_) Å	1.48	1.57	1.62	1.69	1.50	1.62	1.65	1.73
∠(O1′-E-O_*p*_)°	106.08	94.10	106.59	95.64	101.76	87.07	102.72	90.44

**Table 4.** Geometrical values of the Class 2 modelled compounds obtained from M06-2X 6-31+G(2d,2p)/AMBER computation.

geometric	P_*i*_-DNA		As_*i*_-DNA	
parameters	R	TS	R	TS
d(E-O3′) Å	1.64	1.67	1.77	1.78
d(E-O5′) Å	1.63	1.62	1.76	1.75
d(O3′-O5′) Å	2.52	2.47	2.71	2.64
d(E-O_*p*_) Å	1.48	1.59	1.63	1.71
∠(O3′-E-O5′)°	101.00	97.33	100.47	97.23
∠(O_*p*_-E-O3′)°	109.96	95.46	109.87	98.58
∠(O_*p*_-E-O5′)°	105.83	109.43	105.79	109.31

We calculated the rate constant for the hydrolysis of different classes of As/P-esters as shown in table 5. Both Class 1A and 1B dianionic ester of P hydrolyzed much faster than the monoanionic form as reported earlier [12]. We observed the same trend in the case of As esters of Class 1A and Class 1B. Higher stability of monoanionic ester over dianionic ester has biological significance. For instance, a stable molecule, e.g. DNA, with a monoanionic phosphodiester backbone, preserves the genetic information. On the other hand, comparatively facile hydrolyzable Class 1A and 1B esters are available physiologically in dianionic form to provide energetic requirements and assist in several other anabolic processes for an organism [1]. In comparison to the P_*i*_ counterpart of Class 1A and 1B, we found that none of the PASA have reasonable stability against hydrolysis. We calculated the rate for the hydrolysis of As_*i*_-DNA to be 1.23 × 10^−6^ s^−1^ which is slower than other PASA like pyroarsente and ribose-1-arsenate, which have first-order rate constant of 0.08 s^−1^ and 0.07 s^−1^, respectively, in dianionic form and 0.0003 s^−1^ and 0.0007 s^−1^, respectively, in monoanionic form. Higher kinetic stability of As_*i*_-DNA against hydrolysis in comparison to other monoanionic esters of As_*i*_ belonging to Class 1A and 1B, was expected because of steric hindrance and base stacking interactions, [8] but it is still a long way from competing with the stability of P_*i*_-DNA against hydrolysis which has a first-order rate constant value of 5.74 × 10^−20^ s^−1^. Another aspect of selecting P_*i*_ over As_*i*_ is that P_*i*_ is 10^6^ times more stable in monoanionic diester form than in dianionic monoester variants of Class 1B, whereas this difference was found to be narrower in the case of As_*i*_. This difference might favour the integration of dianionic monoesters of P_*i*_ into a relatively more stable biomolecule like DNA. We also found that there is no significant difference among the rate constants of monoanionic pyro-As_*i*_ and ribose-1-As_*i*_, monoionic pyro-P_*i*_ and ribose-1-P_*i*_, similar to the dianionic species of both the esters, which signifies that the conserved sites through which these two esters bonded covalently have nothing to do with the kinetics of the hydrolysis. However, these conserved sites have well-defined regulatory roles in protein–protein interactions [23].

**Table 5.** Values of rate constant (k) (in s^−1^) for the hydrolysis of As/P-esters belonging to different classes.

	Class 1A		Class 1B		Class 2	
anion	Pyro-As_*i*_	Pyro-P_*i*_	Ribose-1-As_*i*_	Ribose-1-P_*i*_	As_*i*_-DNA	P_*i*_-DNA
Mono	0.0003	5.49 × 10^−16^	0.0007	1.58 × 10^−16^	1.23 × 10^−6^	5.74 × 10^−20^
Di	0.08	3.17 × 10^−15^	0.07	8.53 × 10^−14^		

We reported that the different classes of PASA have much less stability against hydrolysis as compared to their P_*i*_ analogue species. Now, the rapid hydrolysis of PASA results in free As_*i*_ which is again going to compete with P_*i*_. In order to avoid the structural confusion between As_*i*_ and P_*i*_, the living organisms require metabolic machineries for the methylation of As_*i*_ that results into organoarsenical compounds which have one or more As-C bonds instead of As-O bonds. Methylation not only resolves the structural ambiguity between As_*i*_ and P_*i*_, it might also prevent conversion of As_*i*_ into a highly reactive form which is known as Arsenite(III). On the other hand, organoarsenicals are highly stable against oxidation as well as against hydrolysis [24], as a result a number of marine organisms accept arsenolipids as a component of their cell membrane which are functionally, but not structurally, similar to phospholipids [25–27]. Arsenolipids primarily consist of As-C bonds which indicates the significant biological stability of As-C bond-based compounds as compared to kinetically unstable As-O bonds containing PASA.

## 4. Conclusion

Our study concluded that all classes of PASA have a higher hydrolysis rate compared to their respective P_*i*_-analogues. In biological systems, where the rate of hydrolysis of P_*i*_ biomolecules is synchronously coupled with another biological process, any alteration in the kinetics of hydrolysis could have a severe effect on the metabolic activity of a cell. Although As_*i*_-DNA has a higher resistance against hydrolysis compared to PASA of Class 1A and 1B, it is still a kinetically unstable molecule, and organisms would face severe consequences by allowing such a highly unstable molecule to substitute P_*i*_-DNA for its genetic material. The rapid rate of the hydrolysis of dianionic PASAs raised another concern regarding their potential to reach and integrate into DNA. However, the mechanism behind the stability of organoarsenicals needs to be investigated for a further understanding of the metabolic process involving methylation of As_*i*_.

## Supplementary Material

supplmentary-geometry.pdf

## Supplementary Material

irc_movies.zip

## Data accessibility

Our data are deposited at Dryad Digital Repository: https://doi.org/10.5061/dryad.56bq0m1 [28].
