# Correction to: ‘Effect of arsenate substitution on phosphate repository of cell: a computational study’

**DOI:** 10.1098/rsos.182121

**Published:** 2019-01-09

**Authors:** Amit Singh, Kousik Giri

*R. Soc. open sci.* **5**, 181565. (Published Online 23 October 2018). (doi:10.1098/rsos.181565)

There is an error in the second sentence of the Introduction in the published paper. The sentence should read: ‘As(V) is also capable of retaining negative charge in its phosphate analogue arsenate…’.

