# Supplementary material for: Effect of arsenate substitution on phosphate repository of cell: a computational study
Source: R Soc Open Sci. 2018 Nov 21;5(11):181565. doi: 10.1098/rsos.181565 (PMC6281905; doi:10.1098/rsos.181565)
Supplement: supplmentary-geometry.pdf [file rsos181565supp1.pdf]

Cartesian coordinates (a.u) of the geometry-optimised structures of all computed compounds used in this study

Class 1A

1. Monoanionic Pyro-Arsenate (Reactant), $N_{imaginary}$ = 0:

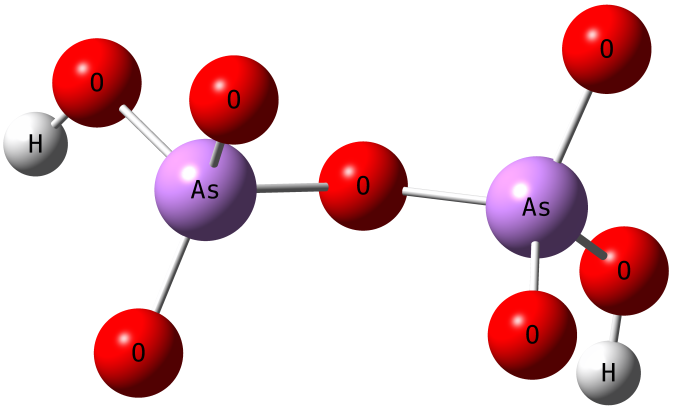

O	-2.524	0.408	-1.328
O	-0.001	-0.190	-0.764
O	1.495	-0.616	1.530
O	2.054	1.594	-0.204
O	2.534	-0.944	-1.020
O	-1.474	1.308	1.037
O	-2.046	-1.469	0.621
H	-2.996	-0.375	-1.629
H	2.472	-1.868	-0.753
As	1.550	0.057	0.035
As	-1.543	-0.010	0.068

2. Monoanionic Pyro-Arsenate (TS1), $N_{imaginary}$ = 1:

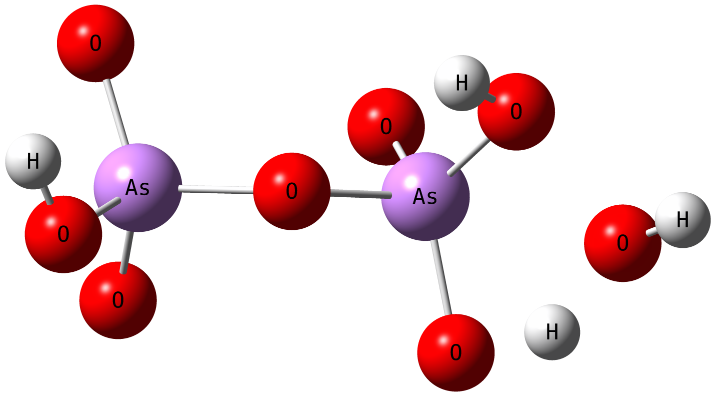

O	2.808	0.298	-1.320
O	0.277	0.519	-0.593
O	-1.104	-0.252	1.628
O	-1.735	-1.134	-1.090
O	-2.016	1.624	-0.371
H	-2.812	-1.059	-0.718
O	-3.575	-0.472	0.156
H	-4.071	0.230	-0.274
O	1.731	-1.714	0.180
O	2.282	0.861	1.314
H	3.259	1.134	-1.159
H	-1.299	2.197	-0.670
As	-1.320	0.072	0.043
As	1.792	-0.082	0.065

3. Dianionic Pyro-Arsenate (Reactant), $N_{imaginary}$ = 0:

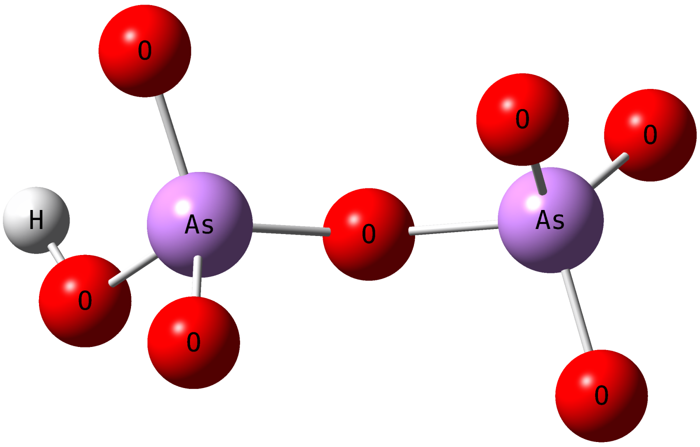

O	-2.064	1.537	-0.439
O	0.005	-0.254	-0.753
O	1.977	-1.317	0.918
O	1.577	1.504	0.744
O	2.560	0.071	-1.360
As	1.512	0.011	0.064
As	-1.633	-0.013	-0.013
O	-1.404	-0.238	1.621
O	-2.530	-1.196	-0.762
H	2.989	-0.786	-1.458

4. Dianionic Pyro-Arsenate (TS1), $N_{imaginary}$ = 1:

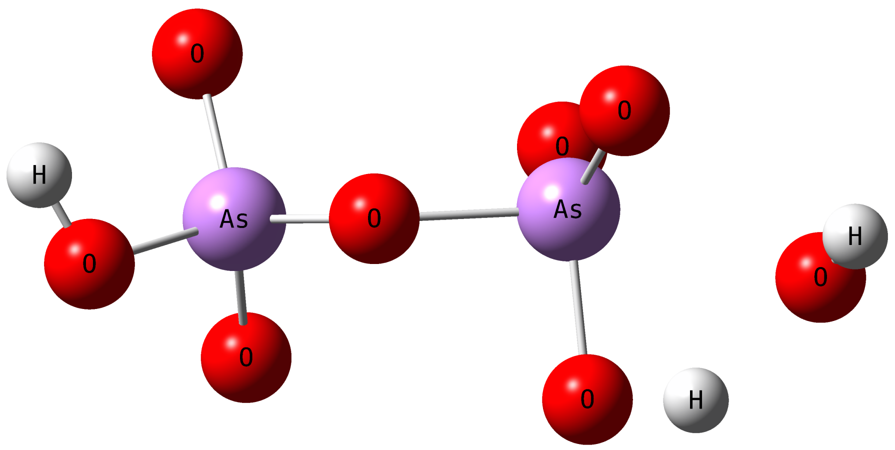

O	-2.858	-0.149	-1.292
O	-0.297	-0.406	-0.708
O	1.030	0.021	1.584
O	1.639	1.262	-0.961
O	2.078	-1.567	-0.614
H	2.593	1.384	-0.679
As	1.376	-0.196	-0.016
O	3.615	0.618	0.349
H	4.059	-0.083	-0.137
As	-1.761	0.077	0.082
O	-1.797	1.688	0.405
O	-2.245	-1.013	1.218
H	-3.296	-1.000	-1.192

5. Monoanionic Pyro-Phosphate (Reactant), $N_{imaginary}$ = 0:

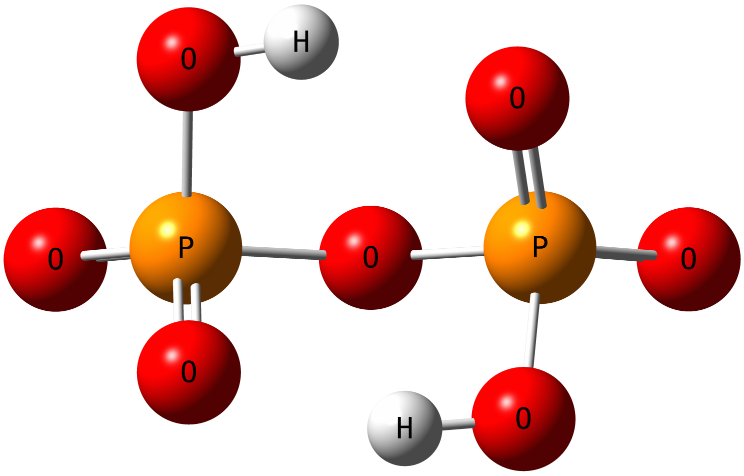

O	-2.529	0.147	1.065
O	-0.000	-0.000	0.922
O	1.241	-1.169	-0.941
O	2.529	-0.147	1.065
O	1.431	1.360	-0.630
O	-1.431	-1.360	-0.630
O	-1.241	1.169	-0.941
P	-1.414	0.083	0.087
P	1.414	-0.083	0.088
H	0.514	1.520	-0.954
H	-0.514	-1.519	-0.954

6. Monoanionic Pyro-Phosphate (TS1), $N_{imaginary}$ = 1:

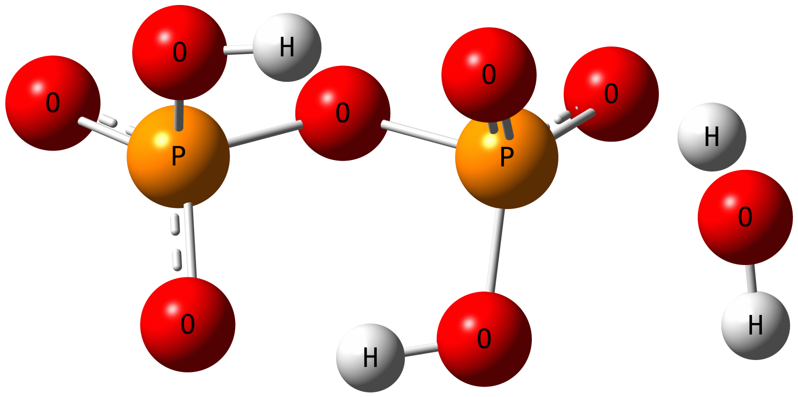

O	-2.918	0.046	-0.971
O	-0.383	0.184	-0.879
O	0.949	1.170	0.970
O	1.921	0.389	-1.422
O	1.031	-1.403	0.353
H	2.843	0.211	-0.939
O	3.238	-0.103	0.405
H	3.394	-1.047	0.499
O	-1.757	1.156	0.975
O	-1.545	-1.381	0.694
P	-1.756	-0.084	-0.057
P	1.104	0.127	-0.095
H	0.058	-1.606	0.514
H	-0.816	1.342	1.198

7. Dianionic Pyro-Phosphate (Reactant), $N_{imaginary}$ = 0:

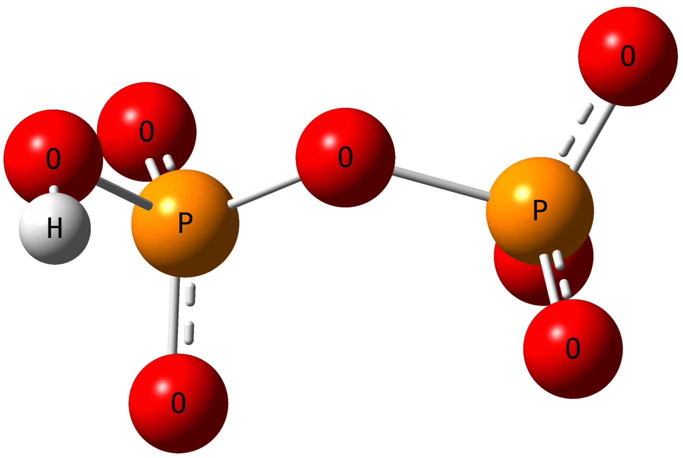

O	-2.360	0.377	-1.251
O	0.006	-0.168	-0.662
O	1.504	-0.665	1.366
O	1.819	1.513	-0.004
O	2.391	-0.687	-1.012
O	-1.459	1.086	1.038
O	-1.896	-1.385	0.534
H	2.326	-1.639	-0.888
P	1.409	0.076	0.066
P	-1.567	-0.005	-0.012

8. Dianionic Pyro-Phosphate (TS1), $N_{imaginary}$ = 1:

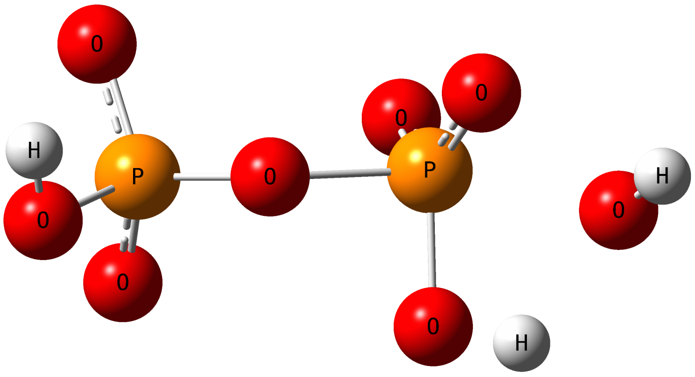

O	-2.751	0.038	-1.188
O	-0.375	-0.292	-0.602
O	1.041	-0.156	1.470
O	1.392	1.278	-0.777
O	1.794	-1.386	-0.786
H	2.329	1.496	-0.628
O	3.318	0.345	0.318
H	3.741	-0.291	-0.263
O	-1.828	1.455	0.633
O	-2.190	-1.077	1.018
H	-2.885	-0.868	-1.481
P	-1.737	0.046	0.123
P	1.312	-0.179	-0.011

Class 1B

1. Monoanionic Ribose-1-Arsenate (Reactant), $N_{imaginary}$ = 0:

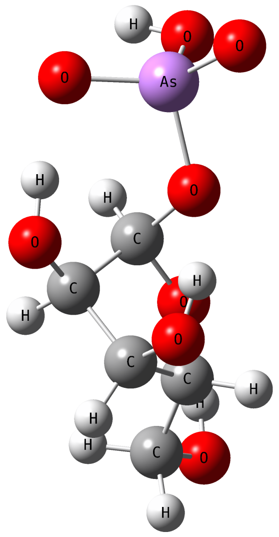

O	-1.630	-1.052	-0.020
O	0.437	-0.253	0.547
O	-1.363	1.873	1.337
O	-0.071	1.982	-1.114
O	-4.305	-1.714	0.014
O	2.416	-1.874	-0.382
O	2.967	0.069	1.493
O	2.275	0.725	-1.230
C	-2.005	1.294	0.223
C	-1.032	0.990	-0.933
C	-2.575	-0.099	0.520
C	-0.508	-0.388	-0.507
C	-3.913	-0.365	-0.144
H	-2.792	1.987	-0.079
H	-1.624	0.835	-1.844
H	-2.649	-0.260	1.598
H	-0.088	-0.984	-1.321
H	-3.856	-0.099	-1.207
H	-4.682	0.251	0.325
H	-0.511	1.431	1.445
H	0.819	1.566	-1.238
H	-3.587	-2.260	-0.326
As	2.148	-0.218	0.117
H	2.489	-1.916	-1.342

2. Monoanionic Ribose-1-Arsenate (TS1), $N_{imaginary}$ = 1:

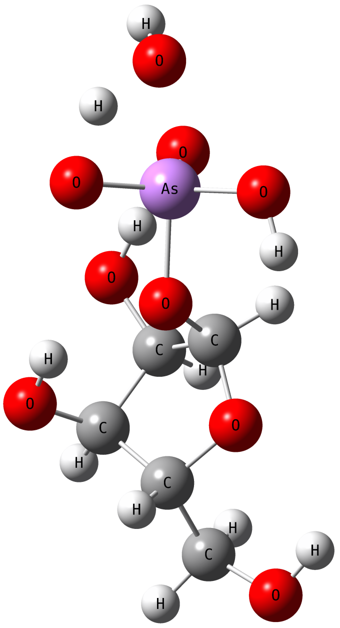

O	-1.922	-1.050	-0.108
O	0.164	-0.306	0.456
O	-1.538	1.783	1.411
O	-0.348	2.014	-1.086
O	-4.601	-1.668	-0.006
O	2.100	-1.949	-0.143
O	2.416	0.414	1.517
O	1.871	0.632	-1.388
C	-2.239	1.283	0.293
C	-1.318	1.031	-0.917
C	-2.822	-0.117	0.530
C	-0.794	-0.372	-0.580
C	-4.192	-0.316	-0.090
H	-3.024	2.006	0.067
H	-1.949	0.930	-1.810
H	-2.855	-0.342	1.599
H	-0.405	-0.923	-1.440
H	-4.175	0.019	-1.135
H	-4.930	0.278	0.451
H	-0.696	1.307	1.455
H	0.521	1.568	-1.272
H	-3.901	-2.199	-0.402
H	3.548	0.253	1.035
As	1.908	-0.219	0.026
O	4.044	-0.225	0.046
H	4.325	0.477	-0.550
H	1.275	-2.375	0.118

3. Dianionic Ribose-1-Arsenate (Reactant), $N_{imaginary}$ = 0:

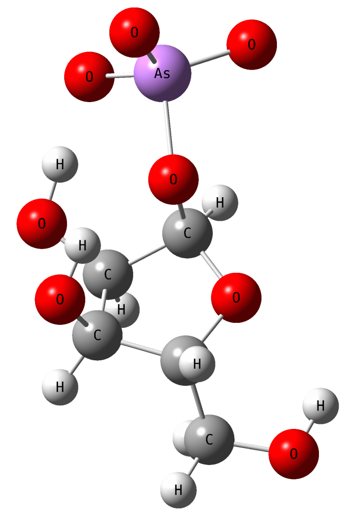

O	-1.643	-1.056	-0.067
O	0.432	-0.283	0.527
O	-1.281	1.805	1.388
O	-0.050	1.981	-1.117
O	-4.317	-1.672	-0.023
O	2.456	-1.827	-0.555
O	2.937	0.035	1.503
O	2.256	0.899	-1.084
C	-1.963	1.286	0.266
C	-1.015	1.004	-0.919
C	-2.555	-0.106	0.524
C	-0.494	-0.386	-0.522
C	-3.910	-0.320	-0.122
H	-2.742	2.007	0.009
H	-1.635	0.869	-1.816
H	-2.615	-0.303	1.598
H	-0.081	-0.968	-1.351
H	-3.870	-0.005	-1.173
H	-4.663	0.282	0.391
H	-0.442	1.324	1.444
H	0.884	1.563	-1.106
H	-3.593	-2.206	-0.370
As	2.183	-0.318	0.073

4. Dianionic Ribose-1-Arsenate (TS1), $N_{imaginary}$ = 1:

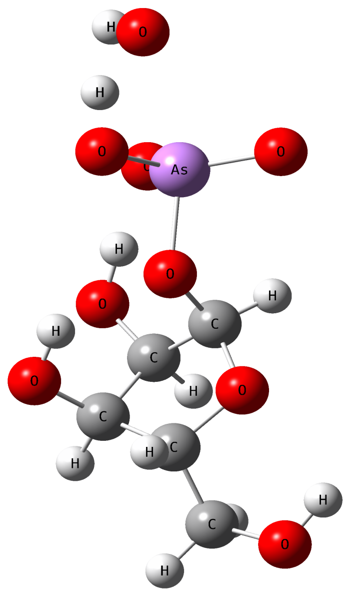

O	-1.939	-1.047	-0.127
O	0.147	-0.288	0.441
O	-1.515	1.769	1.416
O	-0.377	2.023	-1.126
O	-4.612	-1.661	0.021
O	2.092	-1.880	-0.575
O	2.363	-0.001	1.615
O	1.943	0.938	-1.096
C	-2.240	1.283	0.306
C	-1.336	1.039	-0.921
C	-2.820	-0.119	0.541
C	-0.799	-0.361	-0.586
C	-4.204	-0.308	-0.049
H	-3.028	2.010	0.101
H	-1.986	0.934	-1.801
H	-2.832	-0.353	1.609
H	-0.410	-0.914	-1.446
H	-4.211	0.044	-1.089
H	-4.931	0.277	0.518
H	-0.680	1.278	1.432
H	0.549	1.609	-1.103
H	-3.905	-2.185	-0.376
H	3.364	0.091	1.373
As	1.903	-0.341	-0.026
O	4.305	-0.045	0.197
H	4.401	0.809	-0.233

5. Monoanionic Ribose-1-Phosphate (Reactant), $N_{imaginary}$ = 0:

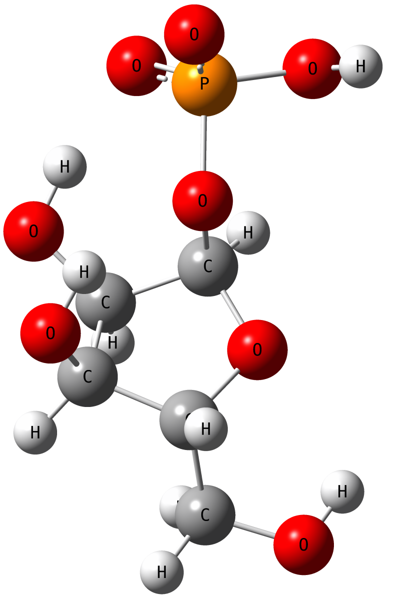

O	-1.271	-1.045	-0.058
O	0.809	-0.282	0.523
O	-0.963	1.830	1.394
O	0.316	2.001	-1.072
O	-3.953	-1.676	-0.042
O	2.574	-1.819	-0.453
O	3.142	-0.149	1.401
O	2.591	0.584	-1.051
C	-1.618	1.296	0.265
C	-0.656	1.017	-0.907
C	-2.203	-0.100	0.517
C	-0.144	-0.378	-0.531
C	-3.546	-0.327	-0.152
H	-2.398	2.008	-0.009
H	-1.256	0.899	-1.818
H	-2.277	-0.297	1.589
H	0.275	-0.952	-1.359
H	-3.488	-0.024	-1.205
H	-4.307	0.281	0.341
H	-0.118	1.370	1.486
H	1.201	1.570	-1.127
H	-3.241	-2.217	-0.402
H	2.640	-2.461	0.263
P	2.401	-0.318	0.130

6. Monoanionic Ribose-1-Phosphate (TS1), $N_{imaginary}$ = 1:

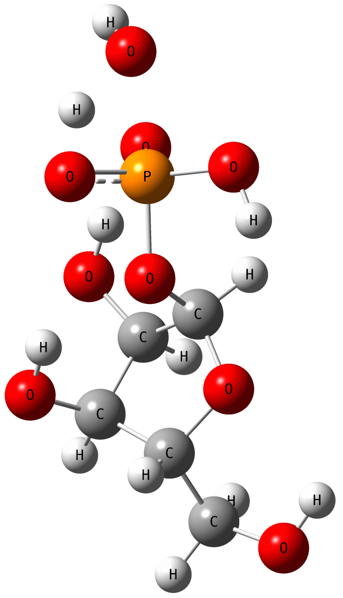

O	-1.594	-1.046	-0.151
O	0.480	-0.278	0.429
O	-1.250	1.777	1.423
O	-0.062	2.054	-1.077
O	-4.265	-1.708	-0.010
O	2.234	-1.868	-0.197
O	2.565	0.216	1.451
O	2.164	0.567	-1.201
C	-1.942	1.275	0.301
C	-1.022	1.056	-0.917
C	-2.498	-0.139	0.521
C	-0.480	-0.345	-0.612
C	-3.875	-0.350	-0.080
H	-2.742	1.985	0.085
H	-1.653	0.968	-1.810
H	-2.508	-0.385	1.586
H	-0.075	-0.873	-1.478
H	-3.879	0.002	-1.120
H	-4.612	0.226	0.482
H	-0.402	1.314	1.469
H	0.815	1.618	-1.180
H	-3.571	-2.224	-0.436
H	1.432	-2.310	0.105
H	3.615	0.081	1.143
O	4.272	-0.405	0.066
H	4.561	0.320	-0.494
P	2.104	-0.278	0.034

7. Dianionic Ribose-1-Phosphate (Reactant), $N_{imaginary}$ = 0:

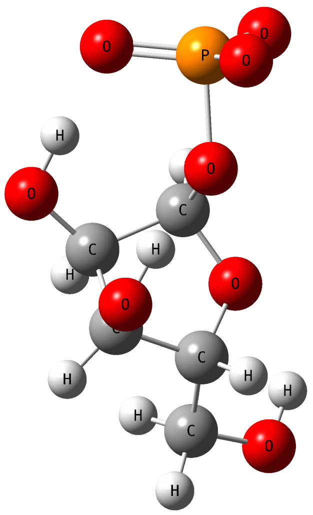

O	-1.309	-1.057	-0.080
O	0.794	-0.357	0.511
O	-0.833	1.770	1.399
O	0.382	1.928	-1.125
O	-4.004	-1.575	-0.041
O	2.618	-1.814	-0.534
O	3.105	-0.238	1.441
O	2.607	0.708	-0.891
C	-1.544	1.291	0.277
C	-0.616	0.985	-0.918
C	-2.185	-0.081	0.522
C	-0.136	-0.423	-0.535
C	-3.549	-0.238	-0.124
H	-2.297	2.044	0.035
H	-1.248	0.880	-1.812
H	-2.252	-0.286	1.594
H	0.260	-1.013	-1.365
H	-3.499	0.089	-1.171
H	-4.278	0.385	0.397
H	-0.021	1.243	1.450
H	1.290	1.480	-1.028
H	-3.297	-2.131	-0.390
P	2.443	-0.454	0.102

8. Dianionic Ribose-1-Phosphate (TS1), $N_{imaginary}$ = 1:

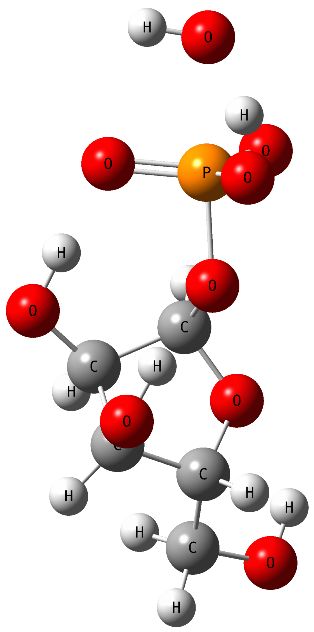

O	-1.612	-1.057	-0.111
O	0.477	-0.279	0.443
O	-1.193	1.733	1.433
O	-0.054	2.012	-1.117
O	-4.281	-1.674	-0.055
O	2.179	-1.764	-0.621
O	2.514	-0.242	1.566
O	2.233	0.872	-0.868
C	-1.923	1.270	0.317
C	-1.018	1.031	-0.910
C	-2.510	-0.131	0.532
C	-0.468	-0.363	-0.573
C	-3.879	-0.317	-0.096
H	-2.707	2.005	0.122
H	-1.665	0.933	-1.792
H	-2.554	-0.368	1.600
H	-0.076	-0.916	-1.430
H	-3.861	0.049	-1.131
H	-4.623	0.257	0.460
H	-0.368	1.221	1.441
H	0.859	1.598	-0.994
H	-3.551	-2.187	-0.422
H	3.497	-0.205	1.480
O	4.354	-0.347	0.034
H	4.513	0.513	-0.363
P	2.160	-0.392	-0.015

Class 2

1. Arsenate-DNA (Reactant), $N_{imaginary}$ = 0:

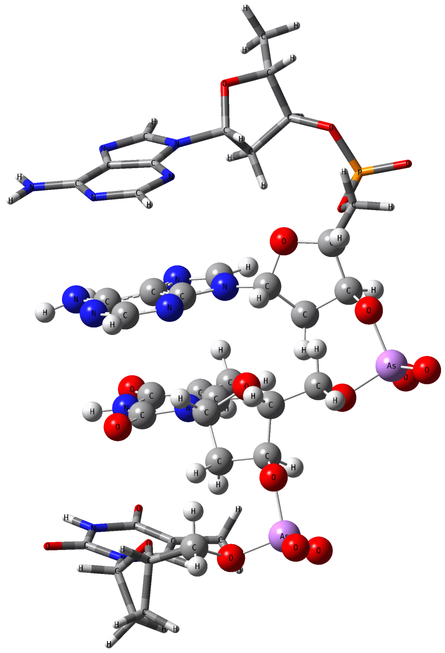

C	10.1682	-0.1718	0.5007
C	8.8529	0.2548	-0.0695
O	8.2585	-0.8916	-0.6737
C	7.8236	0.7673	0.9357
O	7.4445	2.1254	0.6618
C	6.6906	-0.2696	0.8513
C	6.8389	-0.7374	-0.5820
N	3.7258	-3.8349	-3.2441
C	3.8899	-2.5101	-3.2534
N	4.6918	-1.7619	-2.5015
C	5.3916	-2.5454	-1.6432
C	5.3330	-3.9097	-1.5218
C	4.4327	-4.5837	-2.3979
N	4.2807	-5.9379	-2.3905
N	6.1683	-4.3670	-0.5669
C	6.7539	-3.2504	-0.1007
N	6.3278	-2.1160	-0.7387
H	7.4914	-3.2482	0.7016
H	10.6759	0.6801	0.9025
H	9.0848	1.0793	-0.7436
H	8.1932	0.8401	1.9589
H	5.7157	0.1849	1.0266
H	6.3402	-0.0731	-1.2874
H	3.2822	-1.9664	-3.9771
H	3.6320	-6.3764	-3.0275
H	4.8173	-6.5024	-1.7476
H	10.7654	-0.6119	-0.2705
H	6.7515	-1.0656	1.5939
P	6.3819	2.9089	1.5569

C	4.6975	3.3138	-0.4031
O	4.9885	2.6486	0.8318
C	3.3112	2.8540	-0.8628
O	3.3683	1.4395	-1.0351
C	2.1605	3.1399	0.1250
O	1.4051	4.2271	-0.3818
C	1.3948	1.8095	0.1821
C	2.0622	0.9279	-0.8739
N	0.4668	-3.9288	-1.5436
C	0.1558	-2.7991	-2.1943
N	0.6066	-1.5687	-1.9790
C	1.4888	-1.5355	-0.9731
C	1.9025	-2.6186	-0.2036
C	1.3445	-3.8669	-0.5315
N	1.6879	-4.9995	0.1141
N	2.8075	-2.2244	0.7607
C	2.9311	-0.9374	0.5692
N	2.1737	-0.4575	-0.4710
O	6.6554	4.3551	1.4378
O	6.3344	2.3182	2.9081
H	3.5574	-0.2760	1.1473
H	4.6991	4.3954	-0.2675
H	3.0696	3.3399	-1.8144
H	2.5263	3.4013	1.1191
H	0.3337	1.9391	-0.0259
H	1.5159	0.9343	-1.8225
H	-0.5671	-2.9084	-2.9960
H	1.1231	-5.8199	-0.0415
H	2.1634	-4.9222	0.9992
H	5.4398	3.0991	-1.1719
H	1.5067	1.3498	1.1652
C	-1.4273	3.6315	-1.4044
O	-1.2240	3.6743	-0.0005
C	-2.4376	2.5565	-1.7162
O	-1.8829	1.2849	-1.3687
C	-3.7669	2.6897	-0.9485
O	-4.8082	2.5272	-1.9010
C	-3.7027	1.5465	0.0624
C	-2.8672	0.5217	-0.6934
N	-2.1891	-0.4657	0.1289
C	-2.3136	-1.8057	-0.1915
O	-2.9882	-2.2283	-1.1108
N	-1.6082	-2.6484	0.6375
C	-0.7875	-2.3101	1.7068
O	-0.2419	-3.1829	2.3600
C	-0.6590	-0.8777	1.9427
C	-1.3572	-0.0466	1.1459
C	0.2143	-0.4155	3.0675
O	-0.2981	6.2906	0.0369
O	0.2554	4.4120	2.1486
H	-0.1189	-0.8517	4.0119
H	-1.8001	4.5935	-1.7718
H	-2.6531	2.5769	-2.7886
H	-3.8565	3.6606	-0.4590
H	-4.6842	1.1682	0.3437

H	-3.4747	-0.0420	-1.4022
H	-1.6824	-3.6337	0.4118
H	-1.2987	1.0296	1.2619
H	0.1845	0.6708	3.1581
H	-0.4876	3.4005	-1.9197
H	-3.1749	1.8899	0.9534
H	1.2493	-0.7320	2.9119
C	-6.6666	0.0185	-2.0671
O	-6.7717	0.9231	-0.9762
C	-6.9654	-1.4069	-1.5875
O	-6.0547	-1.7472	-0.5405
C	-8.3479	-1.5461	-0.9563
C	-8.0601	-2.1644	0.4232
C	-6.6910	-2.7866	0.1910
N	-5.8669	-2.9567	1.4189
C	-5.3711	-4.1849	1.7426
O	-5.6696	-5.1766	1.0829
N	-4.4854	-4.2829	2.7733
C	-4.1052	-3.1986	3.5150
O	-3.2667	-3.3810	4.3995
C	-4.6619	-1.9514	3.2145
C	-5.5261	-1.8400	2.1251
C	-4.3050	-0.7241	4.0159
O	-7.3592	3.0477	-2.6616
O	-6.5564	3.4125	0.0757
H	-3.5238	-0.1668	3.4992
H	-7.3799	0.2787	-2.8541
H	-6.9403	-2.0278	-2.4835
H	-8.8103	-0.5871	-0.8492
H	-8.8024	-2.9184	0.6863
H	-6.7890	-3.7766	-0.2552
H	-4.0958	-5.1875	2.9975
H	-5.9266	-0.8668	1.8401
H	-3.9474	-1.0236	5.0010
H	-5.6581	0.0391	-2.4928
H	-8.0926	-1.4519	1.2473
H	-5.1872	-0.0942	4.1264
H	-8.9633	-2.1987	-1.5396
H	10.0044	-0.8887	1.2779
As	0.0245	4.7798	0.5722
As	-6.4812	2.6151	-1.3499

2. Arsenate-DNA (TS1), $N_{imaginary}$ = 1:

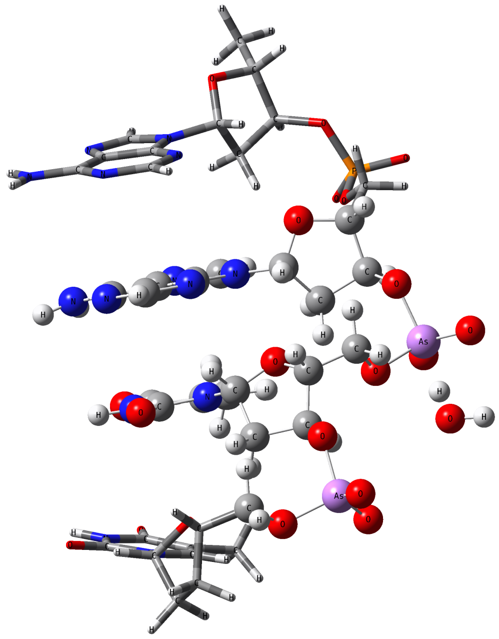

C	10.0525	-0.3481	0.5179
C	8.7513	0.1182	-0.0533
O	8.1390	-1.0017	-0.6886
C	7.7237	0.6289	0.9547
O	7.3747	2.0003	0.7087
C	6.5707	-0.3825	0.8371
C	6.7221	-0.8206	-0.6051
N	3.5702	-3.7923	-3.3632
C	3.7613	-2.4713	-3.3409
N	4.5716	-1.7570	-2.5654
C	5.2477	-2.5739	-1.7194
C	5.1602	-3.9391	-1.6297
C	4.2542	-4.5745	-2.5285
N	4.0747	-5.9251	-2.5533
N	5.9777	-4.4350	-0.6786
C	6.5817	-3.3415	-0.1821
N	6.1844	-2.1845	-0.7976
H	7.3120	-3.3727	0.6262
H	10.5739	0.4839	0.9432
H	9.0058	0.9528	-0.7065
H	8.0857	0.6708	1.9823
H	5.6037	0.0879	1.0147
H	6.2432	-0.1303	-1.2994
H	3.1711	-1.8990	-4.0570
H	3.4228	-6.3357	-3.2055
H	4.5940	-6.5149	-1.9190
H	10.6473	-0.7828	-0.2581
H	6.6088	-1.1961	1.5618
P	6.3204	2.7849	1.6126
C	4.6620	3.2687	-0.3517
O	4.9285	2.5698	0.8701
C	3.2708	2.8929	-0.8533
O	3.2242	1.4756	-1.0135
C	2.1347	3.3008	0.1089
O	1.2842	4.1919	-0.5878

C	1.4849	1.9621	0.4682
C	1.9269	1.0341	-0.6603
N	0.5395	-3.8260	-1.6701
C	0.2967	-2.6817	-2.3253
N	0.6945	-1.4518	-2.0230
C	1.4413	-1.4340	-0.9124
C	1.7996	-2.5385	-0.1461
C	1.2940	-3.7825	-0.5641
N	1.5570	-4.9243	0.1038
N	2.5878	-2.1668	0.9239
C	2.6853	-0.8695	0.8067
N	2.0107	-0.3577	-0.2770
O	6.6243	4.2275	1.5289
O	6.2490	2.1648	2.9495
H	3.2410	-0.2284	1.4737
H	4.6844	4.3466	-0.1914
H	3.0960	3.3838	-1.8149
H	2.5092	3.7947	1.0054
H	0.4027	2.0382	0.5434
H	1.2458	1.0744	-1.5157
H	-0.3144	-2.7790	-3.2170
H	1.0249	-5.7463	-0.1359
H	1.9296	-4.8629	1.0385
H	5.4065	3.0562	-1.1189
H	1.8854	1.6034	1.4182
C	-1.3230	3.1677	-1.5099
O	-1.2572	3.5817	-0.1541
C	-2.0125	1.8334	-1.5888
O	-1.2653	0.8397	-0.8986
C	-3.4020	1.7558	-0.9589
O	-4.3386	2.2645	-1.8845
C	-3.5410	0.2532	-0.6664
C	-2.1100	-0.2860	-0.8426
N	-1.6394	-1.1615	0.2293
C	-1.9624	-2.4949	0.1144
O	-2.6654	-2.9331	-0.7789
N	-1.4253	-3.2932	1.0975
C	-0.6446	-2.8953	2.1775
O	-0.2015	-3.7293	2.9511
C	-0.4417	-1.4581	2.2861
C	-0.9478	-0.6739	1.3138
C	0.3251	-0.9336	3.4590
O	-0.3464	6.2695	-0.6255
O	0.3781	4.7398	1.8349
H	0.4125	0.1521	3.4060
H	-1.9032	3.8848	-2.1003
H	-2.1016	1.5601	-2.6505
H	-3.4211	2.3416	-0.0365
H	-4.1937	-0.2036	-1.4051
H	-2.0175	-0.8647	-1.7642
H	-1.6163	-4.2843	1.0092
H	-0.8305	0.4013	1.3200
H	1.3278	-1.3667	3.4823
H	-0.3229	3.0859	-1.9447
H	-3.9282	0.0502	0.3308

H	-0.1697	-1.1990	4.3960
C	-6.7576	0.2479	-2.1906
O	-6.6390	1.0863	-1.0503
C	-7.0837	-1.1836	-1.7408
O	-6.1893	-1.5661	-0.6943
C	-8.4741	-1.3086	-1.1245
C	-8.2111	-1.9638	0.2428
C	-6.8530	-2.6085	0.0080
N	-6.0433	-2.8233	1.2384
C	-5.5755	-4.0684	1.5382
O	-5.8883	-5.0386	0.8536
N	-4.7012	-4.2079	2.5737
C	-4.3055	-3.1487	3.3432
O	-3.4786	-3.3683	4.2302
C	-4.8341	-1.8839	3.0666
C	-5.6861	-1.7302	1.9729
C	-4.4593	-0.6827	3.8987
O	-6.6781	3.4037	-2.5915
O	-5.8663	3.3701	0.1676
H	-3.6625	-0.1300	3.4015
H	-7.5524	0.6011	-2.8528
H	-7.0632	-1.7843	-2.6505
H	-8.9179	-0.3430	-0.9994
H	-8.9709	-2.7082	0.4825
H	-6.9672	-3.5860	-0.4616
H	-4.3320	-5.1252	2.7804
H	-6.0643	-0.7427	1.7068
H	-4.1166	-1.0118	4.8797
H	-5.8241	0.2257	-2.7615
H	-8.2364	-1.2696	1.0827
H	-5.3295	-0.0375	4.0162
H	-9.0975	-1.9350	-1.7277
H	9.8673	-1.0789	1.2771
H	-0.6218	5.1567	2.1584
O	-1.7968	5.5083	1.6922
H	-1.8435	6.4638	1.5942
As	-0.1443	4.8842	0.2144
As	-5.9545	2.6827	-1.3135

3. Phosphate-DNA (Reactant), $N_{imaginary}$ = 0:

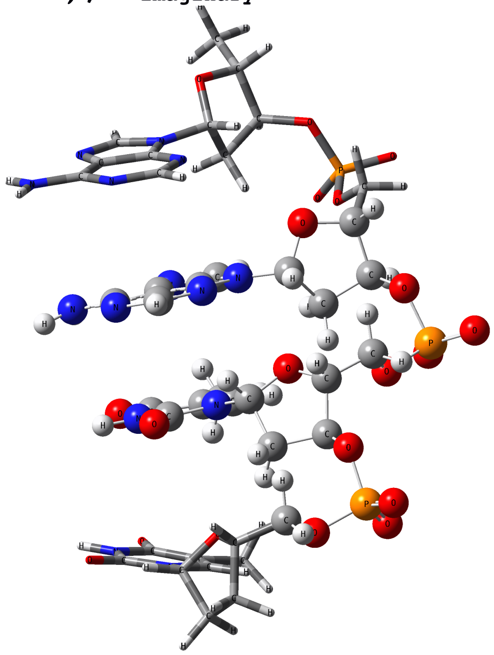

C	9.8104	-0.5328	0.6128
C	8.5417	0.0174	0.0429
O	7.8796	-1.0499	-0.6318
C	7.5305	0.5509	1.0555
O	7.2559	1.9454	0.8474
C	6.3280	-0.3950	0.8962
C	6.4731	-0.7977	-0.5571
N	3.2035	-3.5183	-3.4345
C	3.4625	-2.2103	-3.3705
N	4.2997	-1.5624	-2.5660
C	4.9228	-2.4381	-1.7382
C	4.7637	-3.7989	-1.6904
C	3.8364	-4.3594	-2.6169
N	3.5874	-5.6975	-2.6841
N	5.5434	-4.3646	-0.7466
C	6.1975	-3.3192	-0.2114
N	5.8678	-2.1254	-0.7958
H	6.9158	-3.4123	0.6028
H	10.3693	0.2579	1.0682
H	8.8467	0.8565	-0.5823
H	7.8823	0.5436	2.0876
H	5.3848	0.1197	1.0781
H	6.0387	-0.0632	-1.2350
H	2.9111	-1.5871	-4.0750
H	2.9227	-6.0540	-3.3549
H	4.0681	-6.3320	-2.0627
H	10.3908	-0.9748	-0.1699
H	6.3156	-1.2306	1.5964
P	6.2334	2.7568	1.7640
C	4.6249	3.3842	-0.2012
O	4.8408	2.6366	1.0016
C	3.2303	3.0961	-0.7519
O	3.1113	1.6865	-0.9507
C	2.0763	3.5430	0.1665
O	1.2494	4.4164	-0.5909

C	1.3966	2.2261	0.5381
C	1.7975	1.2931	-0.6001
N	0.2196	-3.4944	-1.6768
C	0.0212	-2.3317	-2.3141
N	0.4661	-1.1227	-1.9942
C	1.2114	-1.1495	-0.8827
C	1.5209	-2.2771	-0.1293
C	0.9725	-3.4956	-0.5684
N	1.1928	-4.6553	0.0813
N	2.3211	-1.9520	0.9468
C	2.4733	-0.6587	0.8456
N	1.8248	-0.1057	-0.2333
O	6.6125	4.1835	1.7267
O	6.1145	2.1022	3.0809
H	3.0537	-0.0497	1.5217
H	4.7013	4.4543	-0.0084
H	3.1218	3.6137	-1.7090
H	2.4197	4.0731	1.0549
H	0.3193	2.3392	0.6286
H	1.1145	1.3712	-1.4515
H	-0.5923	-2.3916	-3.2077
H	0.6469	-5.4618	-0.1779
H	1.5915	-4.6289	1.0063
H	5.3662	3.1560	-0.9671
H	1.8062	1.8570	1.4806
C	-1.4281	3.5800	-1.5184
O	-1.1884	3.9223	-0.1648
C	-2.1777	2.2780	-1.5969
O	-1.4402	1.2388	-0.9625
C	-3.5466	2.2250	-0.9212
O	-4.4945	2.8221	-1.7809
C	-3.7346	0.7148	-0.7190
C	-2.3162	0.1393	-0.8845
N	-1.8733	-0.7264	0.2103
C	-2.2527	-2.0473	0.1350
O	-2.9824	-2.4796	-0.7398
N	-1.7385	-2.8414	1.1342
C	-0.9377	-2.4470	2.2008
O	-0.5302	-3.2763	2.9989
C	-0.6751	-1.0173	2.2681
C	-1.1610	-0.2391	1.2799
C	0.1133	-0.4900	3.4257
O	-0.3975	6.2726	-0.7393
O	0.1015	5.1545	1.5739
H	0.2482	0.5888	3.3381
H	-2.0260	4.3539	-2.0104
H	-2.3184	2.0368	-2.6612
H	-3.5178	2.7571	0.0326
H	-4.3713	0.3261	-1.5094
H	-2.2379	-0.4652	-1.7907
H	-1.9781	-3.8243	1.0789
H	-1.0100	0.8315	1.2610
H	1.0965	-0.9652	3.4664
H	-0.4797	3.4811	-2.0567
H	-4.1636	0.4604	0.2482

H	-0.3933	-0.7021	4.3701
C	-6.9004	1.0194	-2.2292
O	-6.6884	1.7821	-1.0498
C	-7.3190	-0.4066	-1.8407
O	-6.4577	-0.8660	-0.7973
C	-8.7210	-0.4771	-1.2423
C	-8.5080	-1.1852	0.1073
C	-7.1825	-1.8927	-0.1333
N	-6.3993	-2.1856	1.0982
C	-6.0001	-3.4617	1.3650
O	-6.3548	-4.3936	0.6484
N	-5.1461	-3.6771	2.4045
C	-4.7050	-2.6632	3.2094
O	-3.9009	-2.9517	4.0976
C	-5.1641	-1.3649	2.9657
C	-5.9944	-1.1348	1.8686
C	-4.7372	-0.2100	3.8373
O	-6.6115	3.9979	-2.3112
O	-5.8444	3.7646	0.1774
H	-3.9071	0.3149	3.3648
H	-7.6815	1.4694	-2.8468
H	-7.3191	-0.9805	-2.7677
H	-9.1155	0.5062	-1.0926
H	-9.3081	-1.8957	0.3169
H	-7.3418	-2.8486	-0.6332
H	-4.8274	-4.6181	2.5872
H	-6.3178	-0.1215	1.6287
H	-4.4233	-0.5852	4.8112
H	-5.9833	0.9636	-2.8229
H	-8.5070	-0.5158	0.9674
H	-5.5741	0.4760	3.9654
H	-9.3690	-1.0522	-1.8702
H	9.5789	-1.2751	1.3478
P	-0.0739	5.0876	0.0992
P	-5.9721	3.2333	-1.2062

4. Phosphate-DNA (TS1), $N_{imaginary}$ = 1:

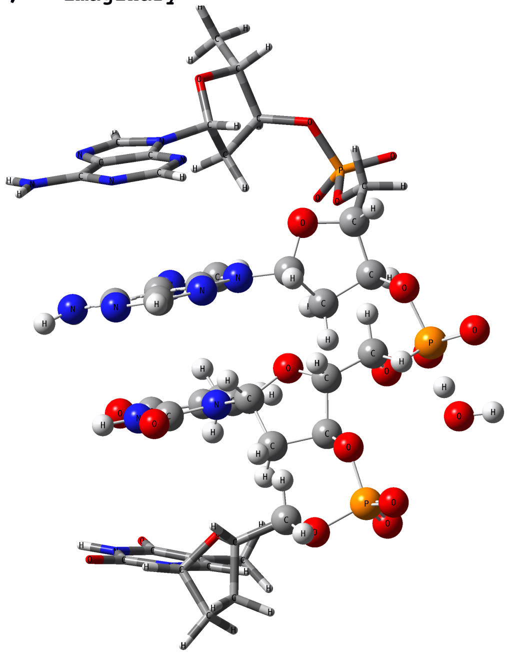

C	9.8243	-0.5351	0.6163
C	8.5534	-0.0076	0.0302
O	7.8981	-1.0964	-0.6161
C	7.5378	0.5476	1.0267
O	7.2558	1.9343	0.7806
C	6.3408	-0.4090	0.8916
C	6.4902	-0.8500	-0.5500
N	3.2395	-3.6657	-3.3567
C	3.4912	-2.3551	-3.3279
N	4.3237	-1.6810	-2.5401
C	4.9505	-2.5304	-1.6881
C	4.7989	-3.8904	-1.6037
C	3.8759	-4.4809	-2.5158
N	3.6344	-5.8217	-2.5470
N	5.5804	-4.4259	-0.6439
C	6.2280	-3.3628	-0.1365
N	5.8925	-2.1871	-0.7534
H	6.9456	-3.4298	0.6808
H	10.3781	0.2708	1.0508
H	8.8545	0.8160	-0.6171
H	7.8883	0.5702	2.0590
H	5.3945	0.1052	1.0584
H	6.0526	-0.1366	-1.2481
H	2.9373	-1.7543	-4.0496
H	2.9726	-6.1999	-3.2087
H	4.1178	-6.4364	-1.9081
H	10.4082	-0.9949	-0.1534
H	6.3321	-1.2253	1.6142
P	6.2276	2.7646	1.6736
C	4.6184	3.3296	-0.3099
O	4.8367	2.6161	0.9130
C	3.2179	3.0137	-0.8377
O	3.1205	1.5974	-1.0103
C	2.0702	3.4585	0.0972
O	1.2302	4.3298	-0.6357

C	1.4156	2.1330	0.4909
C	1.8152	1.1938	-0.6418
N	0.2396	-3.6112	-1.6416
C	0.0550	-2.4620	-2.3075
N	0.5068	-1.2495	-2.0116
C	1.2441	-1.2564	-0.8946
C	1.5466	-2.3708	-0.1185
C	0.9880	-3.5935	-0.5311
N	1.1947	-4.7411	0.1475
N	2.3397	-2.0275	0.9569
C	2.4940	-0.7362	0.8337
N	1.8543	-0.2015	-0.2599
O	6.5989	4.1919	1.5981
O	6.1105	2.1452	3.0076
H	3.0676	-0.1163	1.5052
H	4.6886	4.4049	-0.1460
H	3.0860	3.5133	-1.8014
H	2.4405	3.9819	0.9780
H	0.3382	2.2252	0.5963
H	1.1254	1.2566	-1.4886
H	-0.5530	-2.5384	-3.2035
H	0.6248	-5.5387	-0.0880
H	1.5609	-4.6878	1.0853
H	5.3619	3.0848	-1.0684
H	1.8452	1.7791	1.4300
C	-1.3307	3.3845	-1.5991
O	-1.1479	3.7834	-0.2498
C	-2.0985	2.0910	-1.6436
O	-1.3870	1.0707	-0.9534
C	-3.4858	2.0738	-0.9977
O	-4.4192	2.6200	-1.9062
C	-3.6838	0.5755	-0.7192
C	-2.2738	-0.0201	-0.8789
N	-1.8416	-0.8915	0.2138
C	-2.2198	-2.2124	0.1272
O	-2.9407	-2.6402	-0.7568
N	-1.7161	-3.0111	1.1280
C	-0.9249	-2.6219	2.2035
O	-0.5188	-3.4565	2.9967
C	-0.6661	-1.1920	2.2832
C	-1.1363	-0.4094	1.2915
C	0.1149	-0.6728	3.4493
O	-0.2638	6.2120	-0.8743
O	0.3650	4.9880	1.5195
H	0.2490	0.4066	3.3713
H	-1.9019	4.1422	-2.1452
H	-2.2145	1.8114	-2.7014
H	-3.4785	2.6568	-0.0742
H	-4.3396	0.1553	-1.4768
H	-2.1991	-0.6216	-1.7875
H	-1.9476	-3.9951	1.0606
H	-0.9767	0.6600	1.2755
H	1.0980	-1.1476	3.4905
H	-0.3632	3.2523	-2.0920
H	-4.0966	0.3762	0.2679

H	-0.3969	-0.8940	4.3886
C	-6.8742	0.8393	-2.2974
O	-6.6423	1.6443	-1.1502
C	-7.3021	-0.5706	-1.8606
O	-6.4397	-0.9967	-0.8041
C	-8.7045	-0.6326	-1.2623
C	-8.4896	-1.3027	0.1063
C	-7.1598	-2.0091	-0.1134
N	-6.3767	-2.2642	1.1264
C	-5.9708	-3.5304	1.4282
O	-6.3194	-4.4833	0.7367
N	-5.1171	-3.7128	2.4743
C	-4.6827	-2.6749	3.2519
O	-3.8782	-2.9348	4.1486
C	-5.1486	-1.3863	2.9725
C	-5.9787	-1.1906	1.8687
C	-4.7293	-0.2058	3.8130
O	-6.5164	3.7860	-2.5275
O	-5.7829	3.6774	-0.0190
H	-3.9015	0.3106	3.3276
H	-7.6554	1.2748	-2.9245
H	-7.2978	-1.1694	-2.7718
H	-9.1047	0.3522	-1.1398
H	-9.2859	-2.0117	0.3340
H	-7.3131	-2.9790	-0.5874
H	-4.7934	-4.6466	2.6827
H	-6.3073	-0.1860	1.6009
H	-4.4147	-0.5528	4.7972
H	-5.9619	0.7534	-2.8955
H	-8.4934	-0.6102	0.9479
H	-5.5701	0.4787	3.9215
H	-9.3485	-1.2280	-1.8752
H	9.5958	-1.2584	1.3709
H	-0.4917	5.4069	1.9008
O	-1.7894	5.7684	1.2144
H	-1.8233	6.6951	0.9629
P	-0.1426	5.0262	0.0119
P	-5.9009	3.0754	-1.3740
