# Supplementary figures and images for: Effect of arsenate substitution on phosphate repository of cell: a computational study
Source: R Soc Open Sci. 2018 Nov 21;5(11):181565. doi: 10.1098/rsos.181565 (PMC6281905; doi:10.1098/rsos.181565)

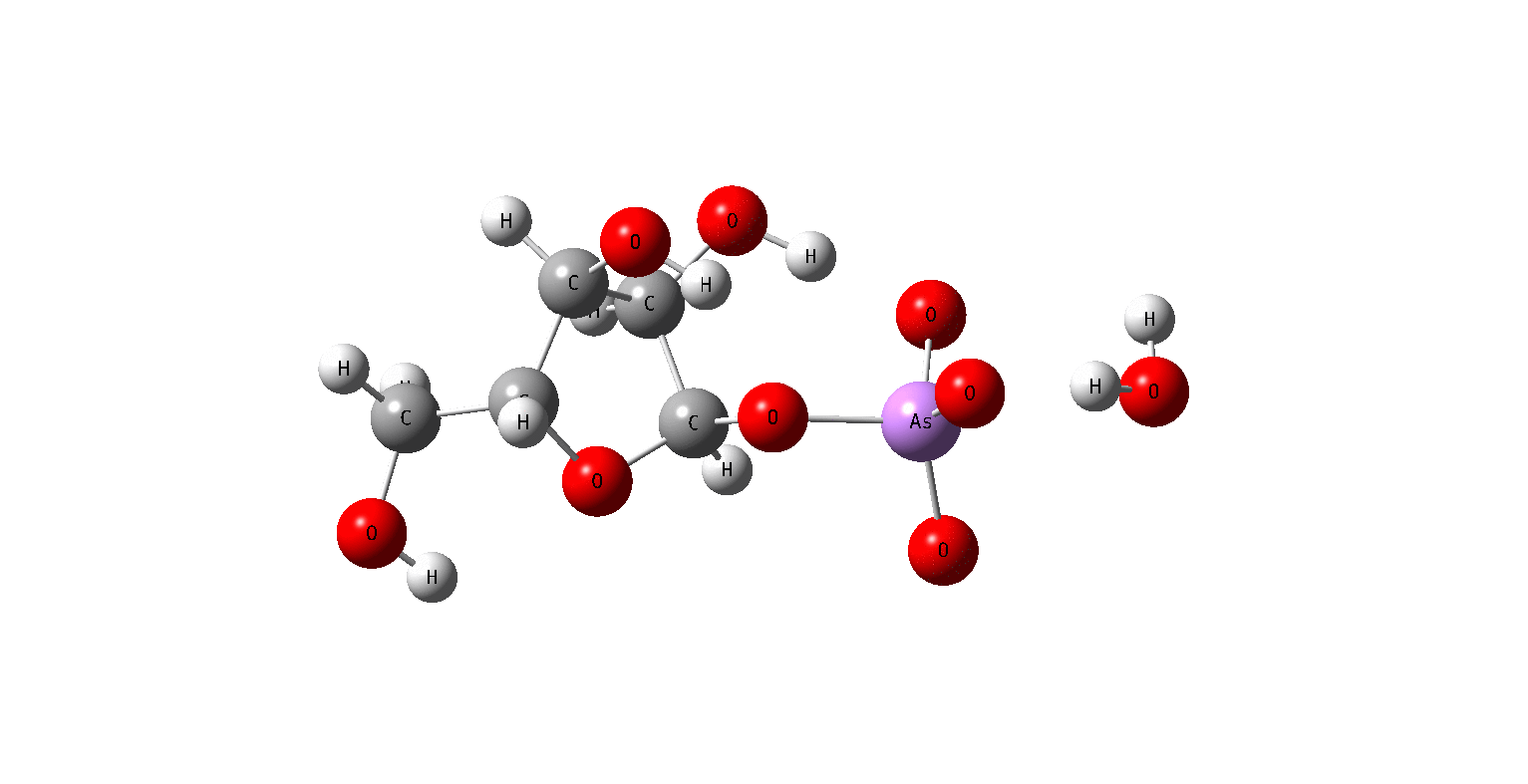

Supplement: irc_movies.zip [file rsos181565supp2.zip › irc_movie/As-1-ribose_dianionic_form_irc.gif]

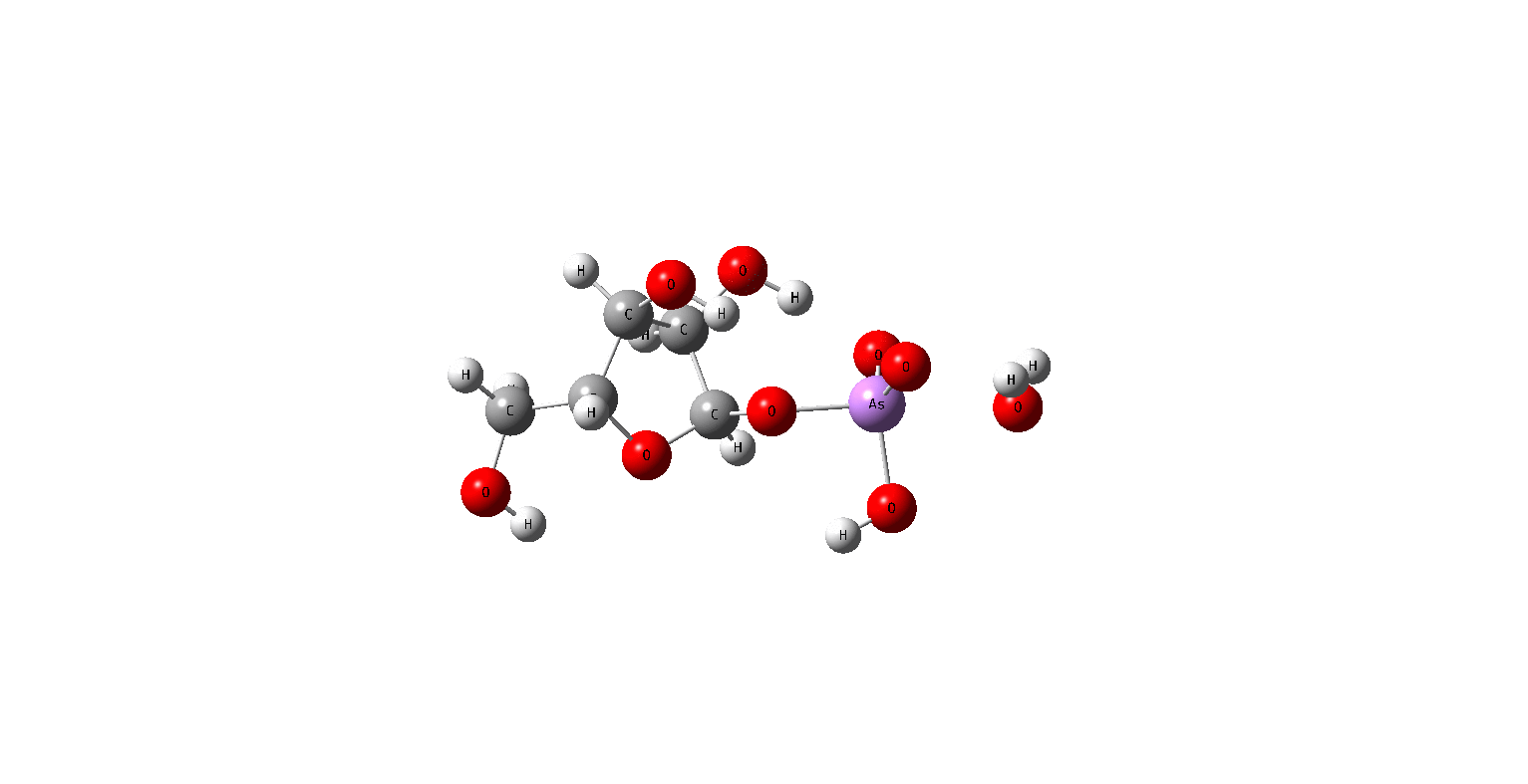

Supplement: irc_movies.zip [file rsos181565supp2.zip › irc_movie/As-1-ribose_monoanionic_form_irc.gif]

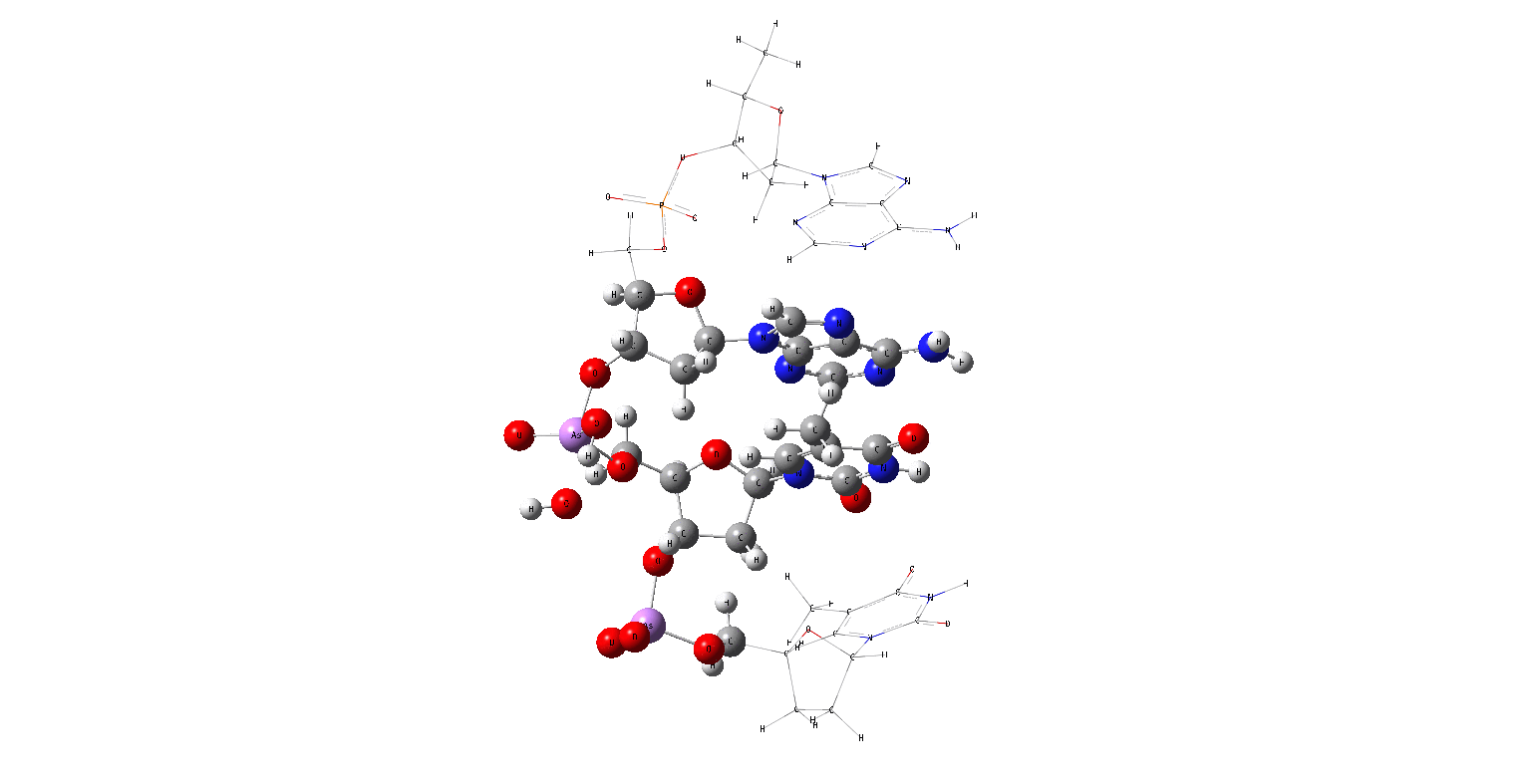

Supplement: irc_movies.zip [file rsos181565supp2.zip › irc_movie/As-DNA_irc.gif]

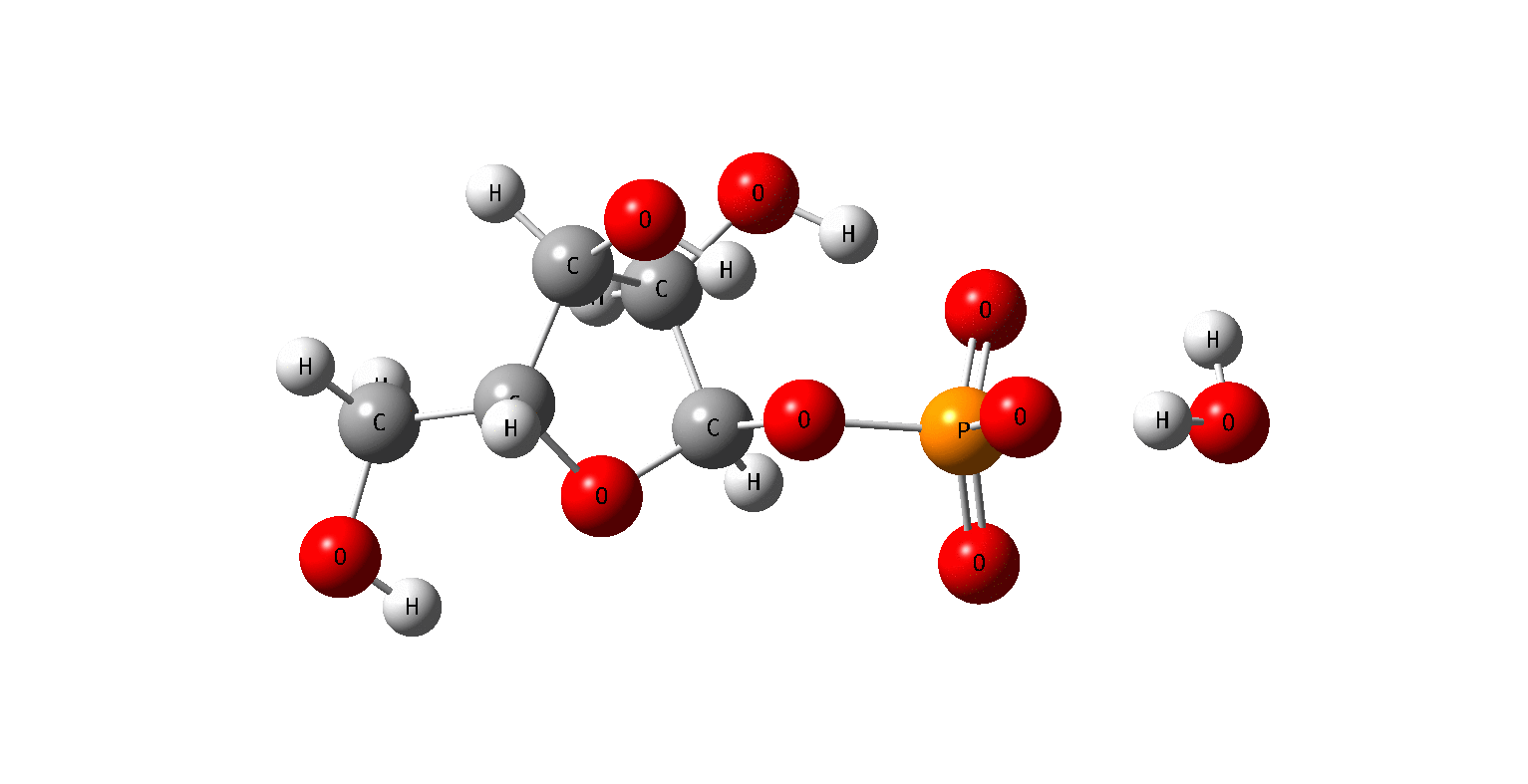

Supplement: irc_movies.zip [file rsos181565supp2.zip › irc_movie/P-1-ribose_dianionic_form_irc.gif]

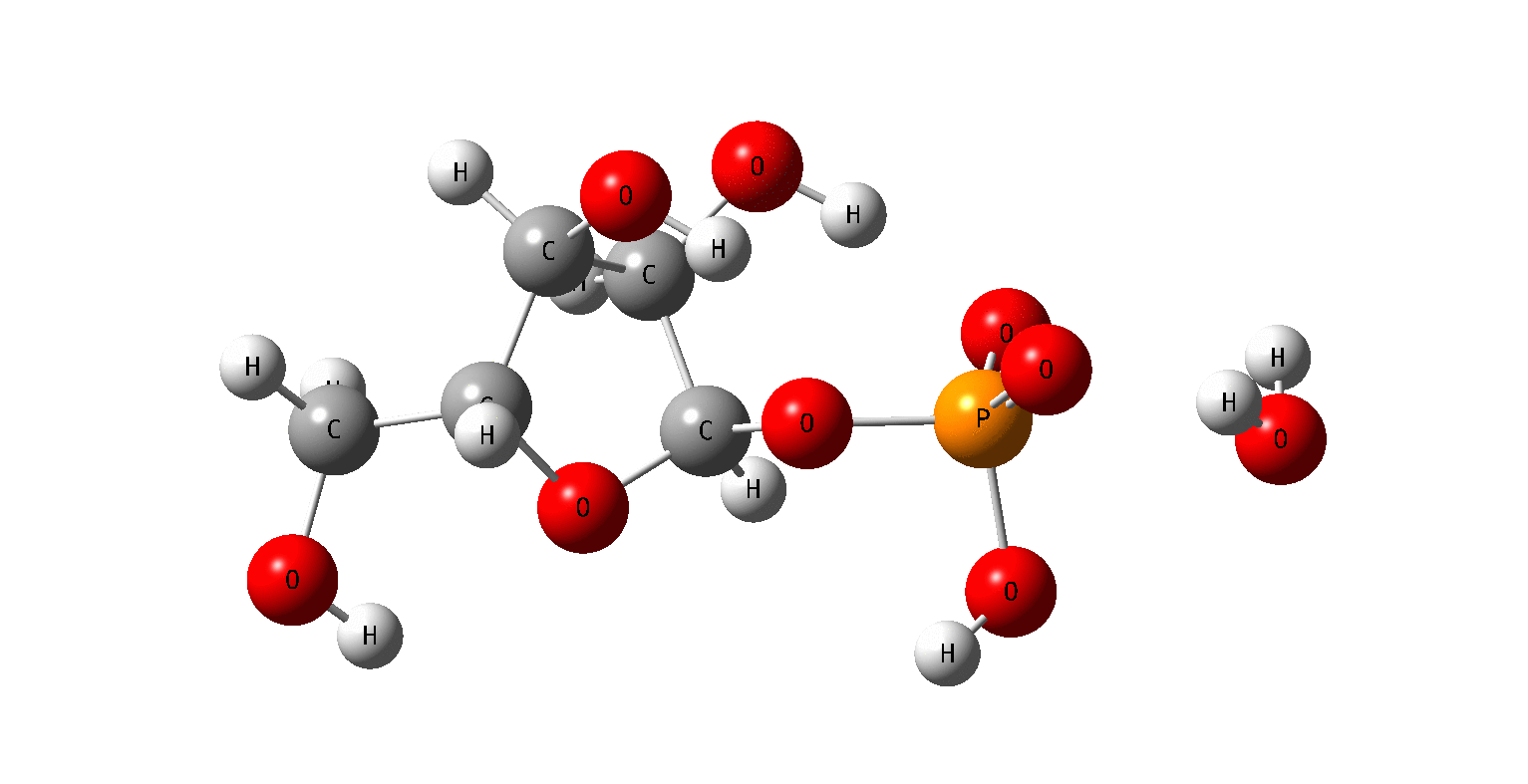

Supplement: irc_movies.zip [file rsos181565supp2.zip › irc_movie/P-1-ribose_monoanionic_form_irc.gif]

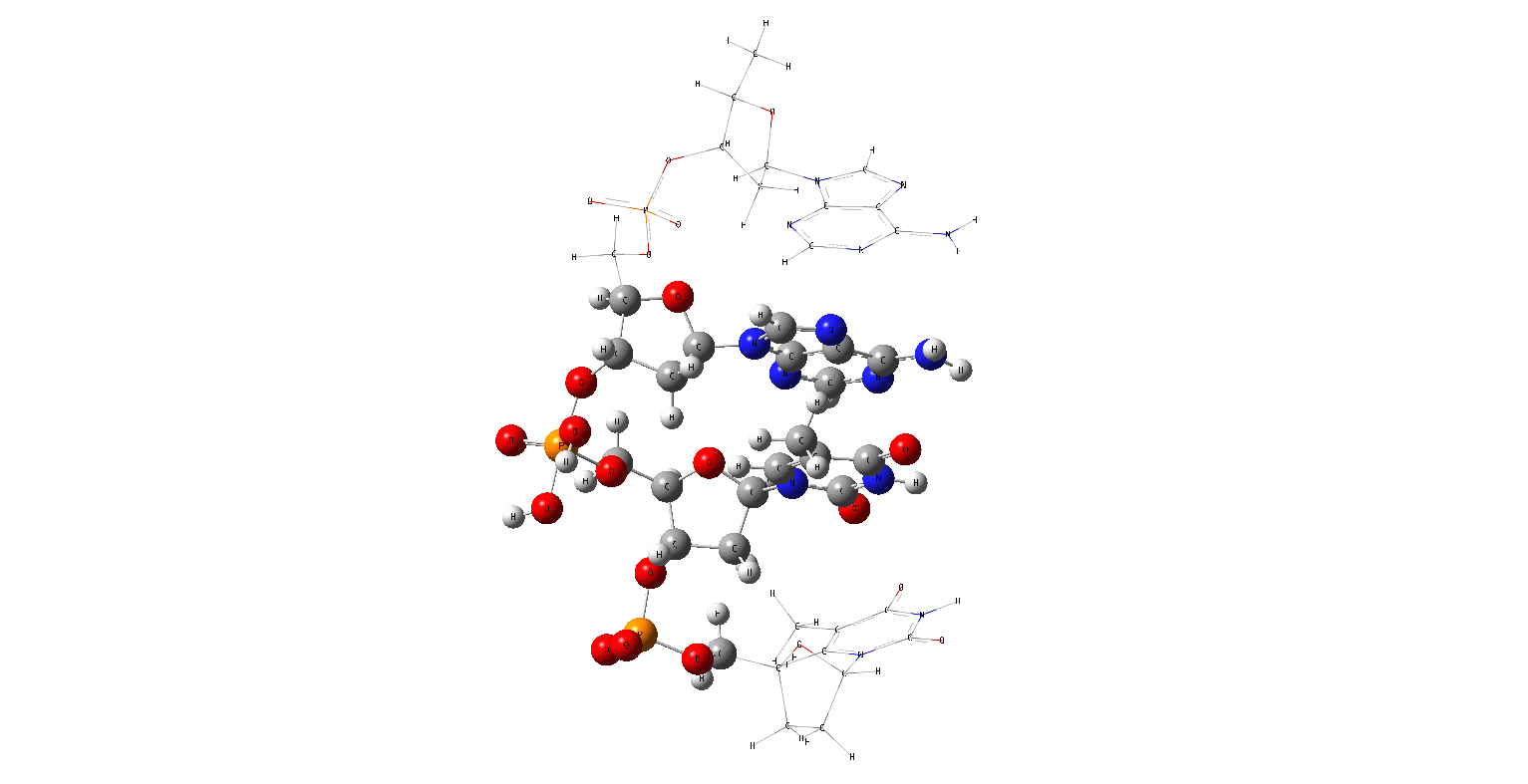

Supplement: irc_movies.zip [file rsos181565supp2.zip › irc_movie/P-DNA_irc.gif]

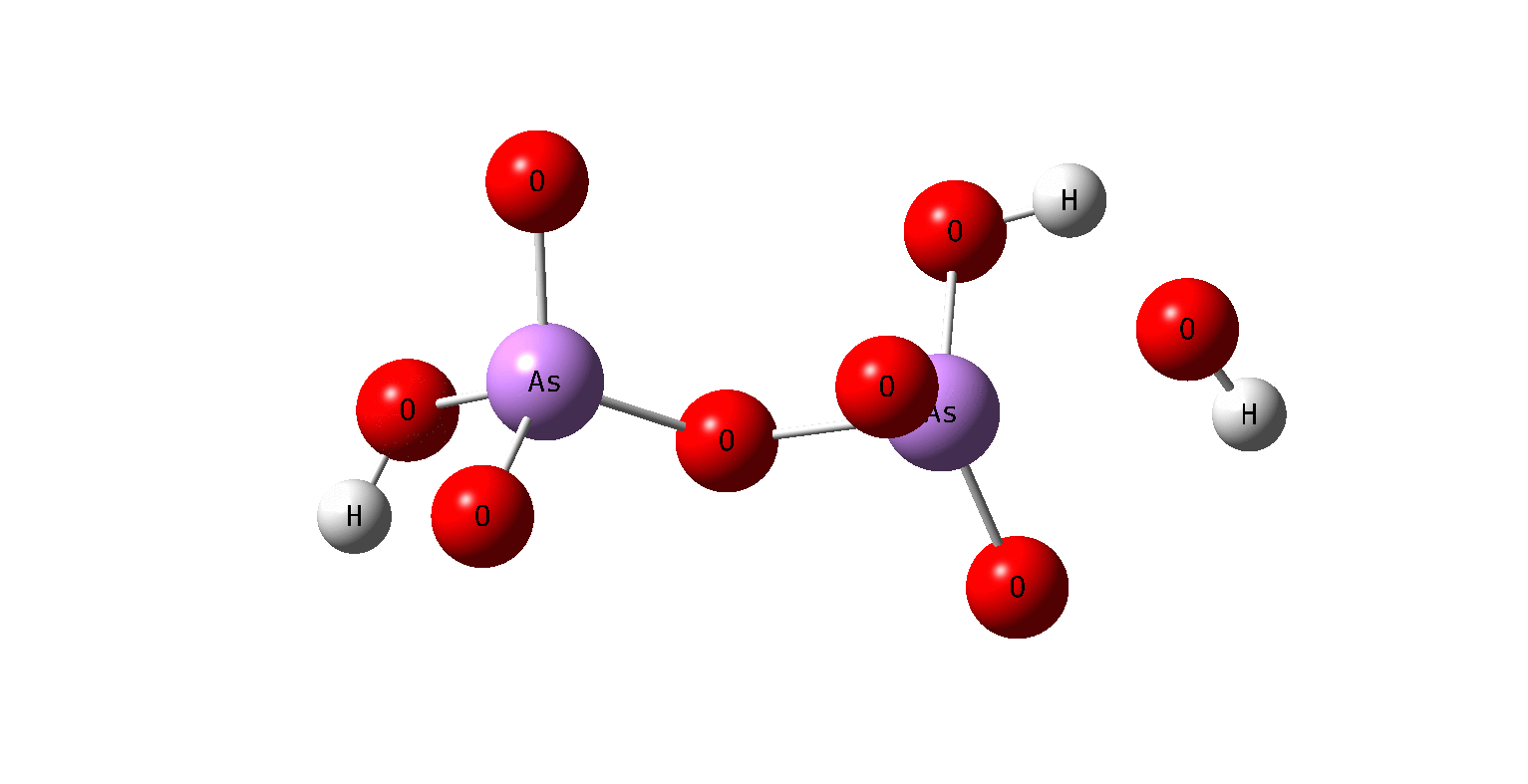

Supplement: irc_movies.zip [file rsos181565supp2.zip › irc_movie/pyro-As_dianionic_form_irc.gif]

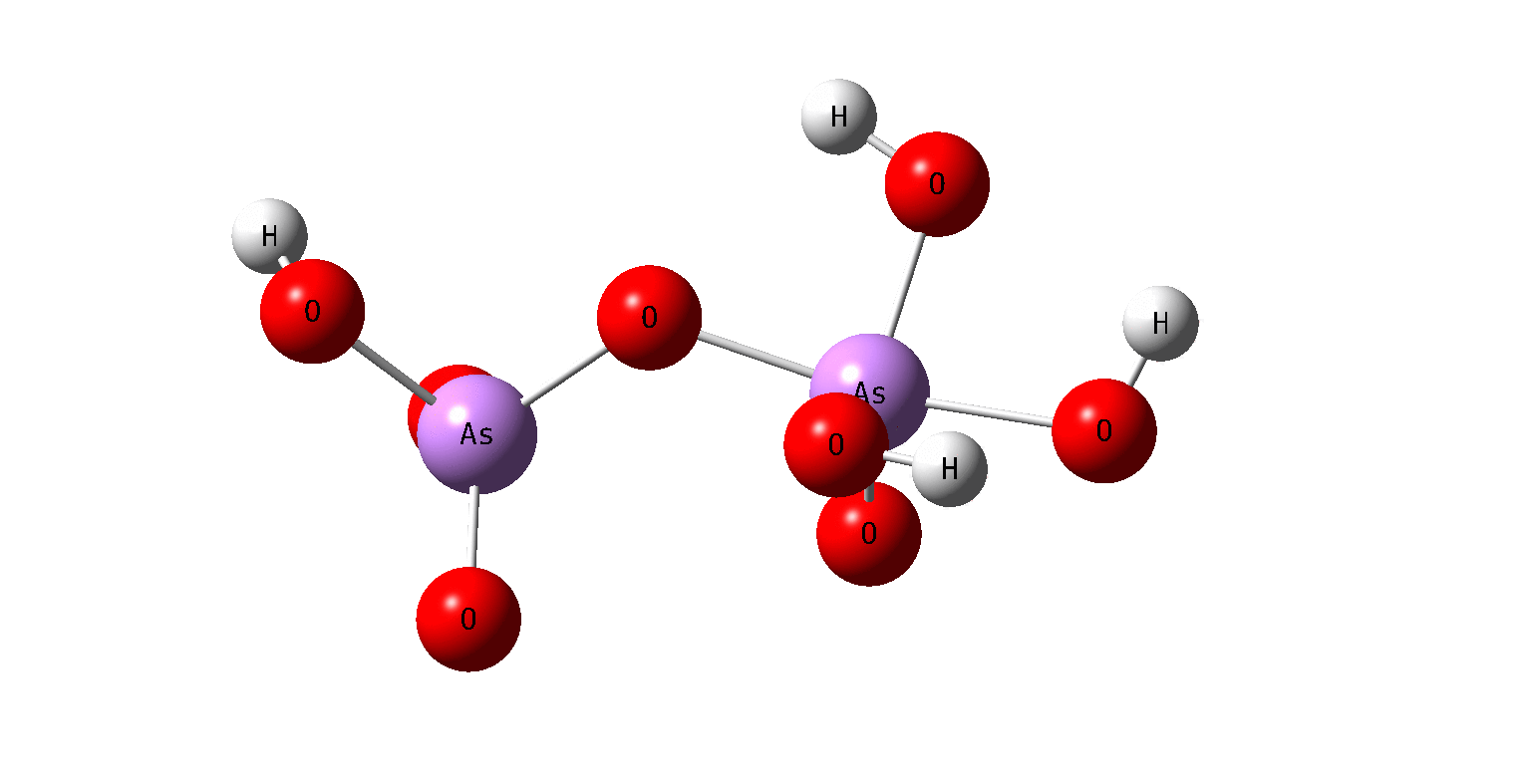

Supplement: irc_movies.zip [file rsos181565supp2.zip › irc_movie/pyro-As_monoanionic_form_irc.gif]

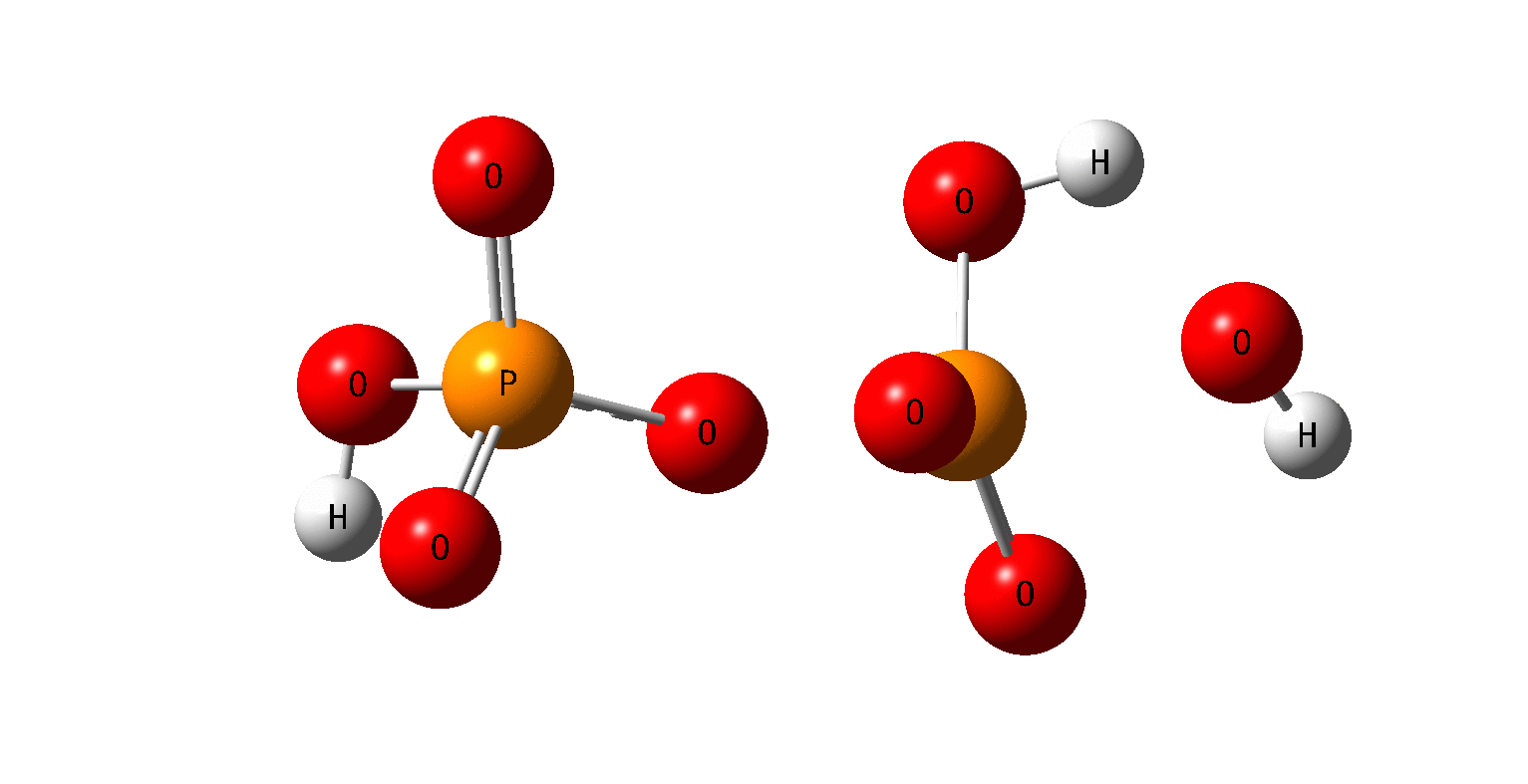

Supplement: irc_movies.zip [file rsos181565supp2.zip › irc_movie/pyro-P_dianionic_form_irc.gif]

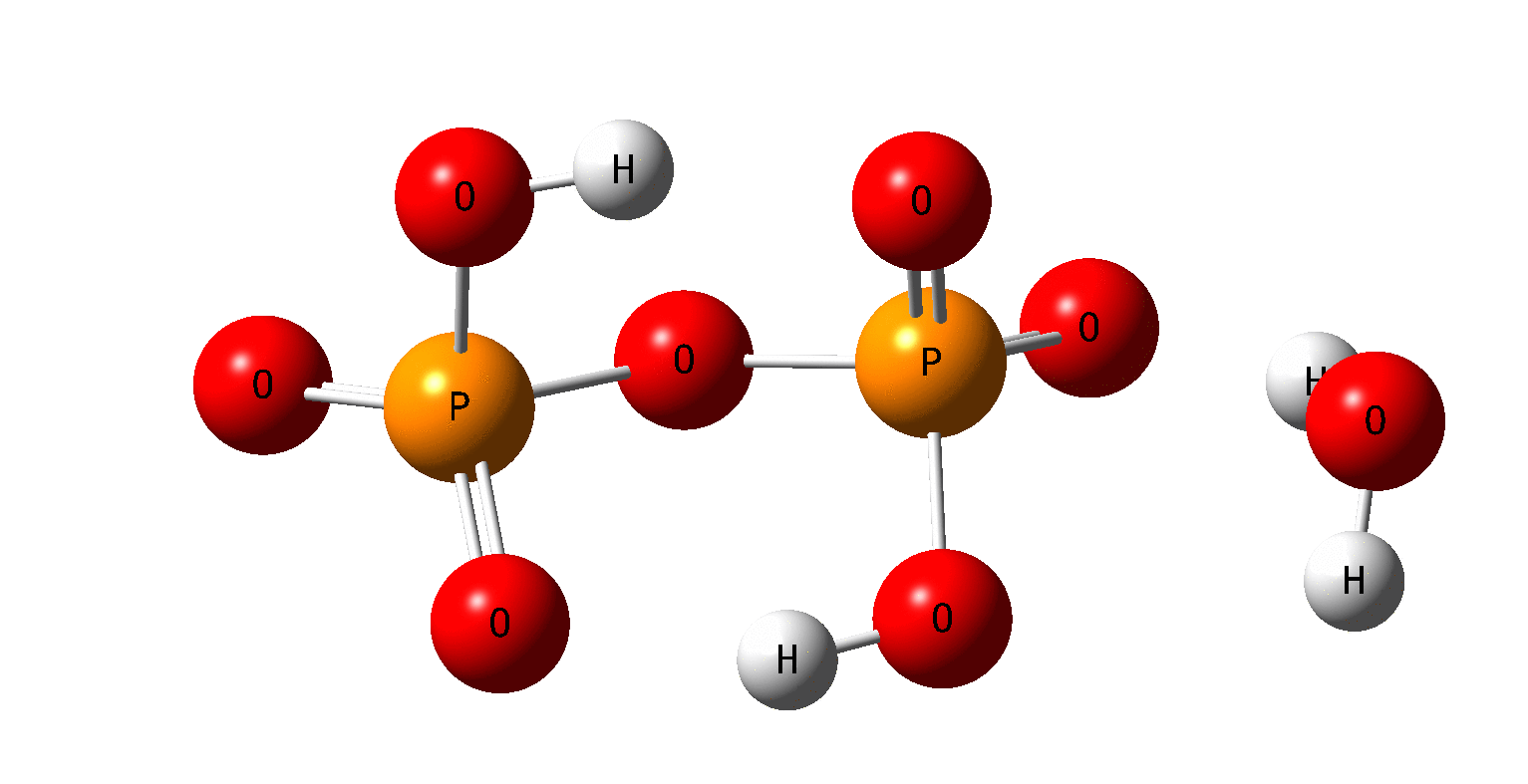

Supplement: irc_movies.zip [file rsos181565supp2.zip › irc_movie/pyro-P_monoanionic_form_irc.gif]
